# Amorphization and modified release of ibuprofen by post-synthetic and solvent-free loading into tailored silica aerogels

**DOI:** 10.1080/10717544.2022.2092237

**Published:** 2022-07-15

**Authors:** Ajmal Zarinwall, Viktor Maurer, Jennifer Pierick, Victor Marcus Oldhues, Julian Cedric Porsiel, Jan Henrik Finke, Georg Garnweitner

**Affiliations:** a Institute for Particle Technology (iPAT), Technische Universität Braunschweig, Braunschweig, Germany; b Center of Pharmaceutical Engineering (PVZ), Technische Universität Braunschweig, Braunschweig, Germany

**Keywords:** Drug release, bioavailability, surface functionalization, supercritical drying, surface modification

## Abstract

Promising active pharmaceutical ingredients (APIs) often exhibit poor aqueous solubility and thus a low bioavailability that substantially limits their pharmaceutical application. Hence, efficient formulations are required for an effective translation into highly efficient drug products. One strategy is the preservation of an amorphous state of the API within a carrier matrix, which leads to enhanced dissolution. In this work, mesoporous silica aerogels (SA) were utilized as a carrier matrix for the amorphization of the poorly water-soluble model drug ibuprofen. Loading of tailored SA was performed post-synthetically and solvent-free, either by co-milling or via the melting method. Thorough analyses of these processes demonstrated the influence of macrostructural changes during the drying and grinding process on the microstructural properties of the SA. Furthermore, interfacial SA-drug interaction properties were selectively tuned by attaching terminal hydrophilic amino- or hydrophobic methyl groups to the surface of the gel. We demonstrate that not only the chemical surface properties of the SA, but also formulation-related parameters, such as the carrier-to-drug ratio, as well as process-related parameters, such as the drug loading method, decisively influence the ibuprofen adsorption efficiency. In addition, the drug-loaded SA formulations exhibited a remarkable physical stability over a period of 6 months. Furthermore, the release behavior is shown to change considerably with different surface properties of the SA matrix. Hence, the reported results demonstrate that utilizing specifically processed and modified SA offers a compelling technique for enhancement of the bioavailability of poorly-water soluble APIs and a versatile adjustment of their release profile.

## Graphical Abstract

**Figure:**
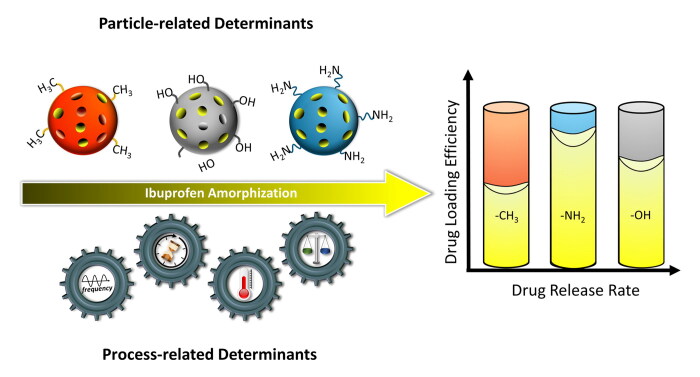

## Introduction

In the recent decades, a steadily growing number of active pharmaceutical ingredients (APIs) with increased selectivity and thus enhanced effects have been developed by the life science industry (Lipinski, 2000). However, these APIs mostly exhibit a poor aqueous solubility which often translates into a low bioavailability of the novel potential drug candidates. Hence, among all recently discovered chemical entities, 90% of the drug candidates are classified into the two low solubility categories of the Biopharmaceutical Classification System, i.e. classes II and IV (Lipp, 2013). In response, the development of innovative galenic strategies to overcome the low solubility is a central and overarching goal of pharmaceutical formulation design. These solubility-enhancing strategies differ depending on the intrinsic properties of the API, the manufacturing processes and the routes of delivery. Despite notable advances in parenteral drug delivery routes (Gulati & Gupta, 2011; Ag Seleci et al., 2016; Bhargava et al., 2017; Marques et al., 2019; Maurer et al., 2021; Zarinwall et al., 2021a; Maurer et al., 2022), the oral route remains the most favored method of administration (Gabor et al., 2010; Williams et al., 2016) because of its high convenience, patient compliance and reduced health care costs in addition to lower production costs (Sastry et al., 2000; Pfeiffer et al., 2006). However, upon oral administration of a solid dosage form, the dissolution rate of the drug in the gastrointestinal tract fluids limits the extent of absorption by the intestinal mucosa and thus its pharmaceutical effect. Consequently, numerous novel formulation approaches have been developed and utilized to overcome this challenge (Singh et al., 2018; Melzig et al., 2018a,b; Suresh et al., 2020; Wewers et al., 2020; Steiner et al., 2021). To this end, the conversion of a thermodynamically stable crystal structure to a high-energy amorphous form of an API constitutes a promising procedure in order to increase its solubility, dissolution rate and thus the oral bioavailability. Since the amorphous state is thermodynamically unstable, the formulation must comprise a stabilizing carrier that prevents recrystallization of the API and consequently preserves the enhanced dissolution properties (Mužík et al., 2020). In particular, mesoporous silica aerogels (SA) belong to the most promising carrier materials for future drug delivery applications owing to their great versatility, chemically inert matrix, large specific surface area (400–1,000 m^2^/g) and high porosity values (90–99%) (Gorle et al., 2009; Hentzschel et al., 2012; García-González et al., 2021). In contrast to many other inorganic materials, amorphous silica was proven not to be harmful to the human body in extensive clinical tests, and is generally recognized as safe for oral delivery. Amorphous SA can be expected to have similar clinical characteristics (Ulker & Erkey, 2014; Diab et al., 2017; Paul et al., 2020). Spatial confinement of APIs inside the pores of the SA matrix can substantially hinder the transformation into the crystalline state and hence tremendously improve the long-term stability of the amorphous drug. In respect thereof, drug loading of synthesized mesoporous silica aerogels is most commonly achieved through supercritical CO_2_ (scCO_2_) impregnation methods (Smirnova et al., 2004b; Gorle et al., 2009; Veres et al., 2015). However, as stated in the literature (Smirnova et al., 2004a,b; 2005), in this method the drug loading efficiency essentially depends on the CO_2_ solubility of the drug molecules and hence lacks versatility regarding the impregnation of different APIs. Moreover, the time-consuming supercritical loading that in turn results in further high economical expenses, represents another significant drawback that needs to be considered for a transfer into a large-scale development.

In contrast, the application of cost-effective and solvent-free drug loading processes surpass those obstacles and allow a more versatile proceeding independently of the solubility characteristics of the drug. In this respect, co-milling represents an attractive and environmentally friendly method that fulfills the aforementioned demands (Bahl & Bogner, 2006; Colombo et al., 2009; Barzegar-Jalali et al., 2010; Grobelny et al., 2015). Here, amorphization is facilitated by the disruption of the molecular arrangement and the creation of structural defects in the crystal lattice of the API while it becomes embedded in the SA excipient at the same time (Boldyrev, 1993). However, the energy introduced into the API-SA carrier blend by co-milling can furthermore cause structural deformation of the SA matrix (Michalchuk et al., 2013; 2014). To ensure the efficient amorphization and loading of the API while keeping structural degradation of the SA to a minimum, a thorough elucidation of crucial process-SA structure correlations is necessary. A less destructive, alternative solvent-free method is loading of the drug in a melted state into the SA matrix (Aerts et al., 2010; Uejo et al., 2013). This procedure entails the physical mixing of a crystalline API and the carrier material, followed by heating the blend above the melting temperature of the drug to commence amorphization. Capillary forces then confine the amorphized API inside the SA pores. However, as any excess drug remaining outside this capillary system potentially facilitates unwanted recrystallization, which may result in unpredictable changes in the dissolution rate and ultimately in the pharmacokinetics of the formulation, a detailed study of the process parameters and the utilized ratio of components is inevitable. To the best of our knowledge, no studies have yet been performed that examine the potential and effects of either strategy for loading self-synthesized SA with APIs. Moreover, defined tuning of the matrix surface by selectively functionalizing the surface of SA cluster particles with different terminal entities allows to specifically adjust the affinity of the API to the matrix and thus its rate of release from the carrier system. In this way, SA-based formulations can be individually processed to enhance the efficacy of different therapeutic applications, e.g. by achieving a relevant dose at the site of action for an extended period of time without causing unwanted side effects of over-exposure through high drug concentrations (Rambhia & Ma, 2015; García-González et al., 2021).

**Figure 1.**
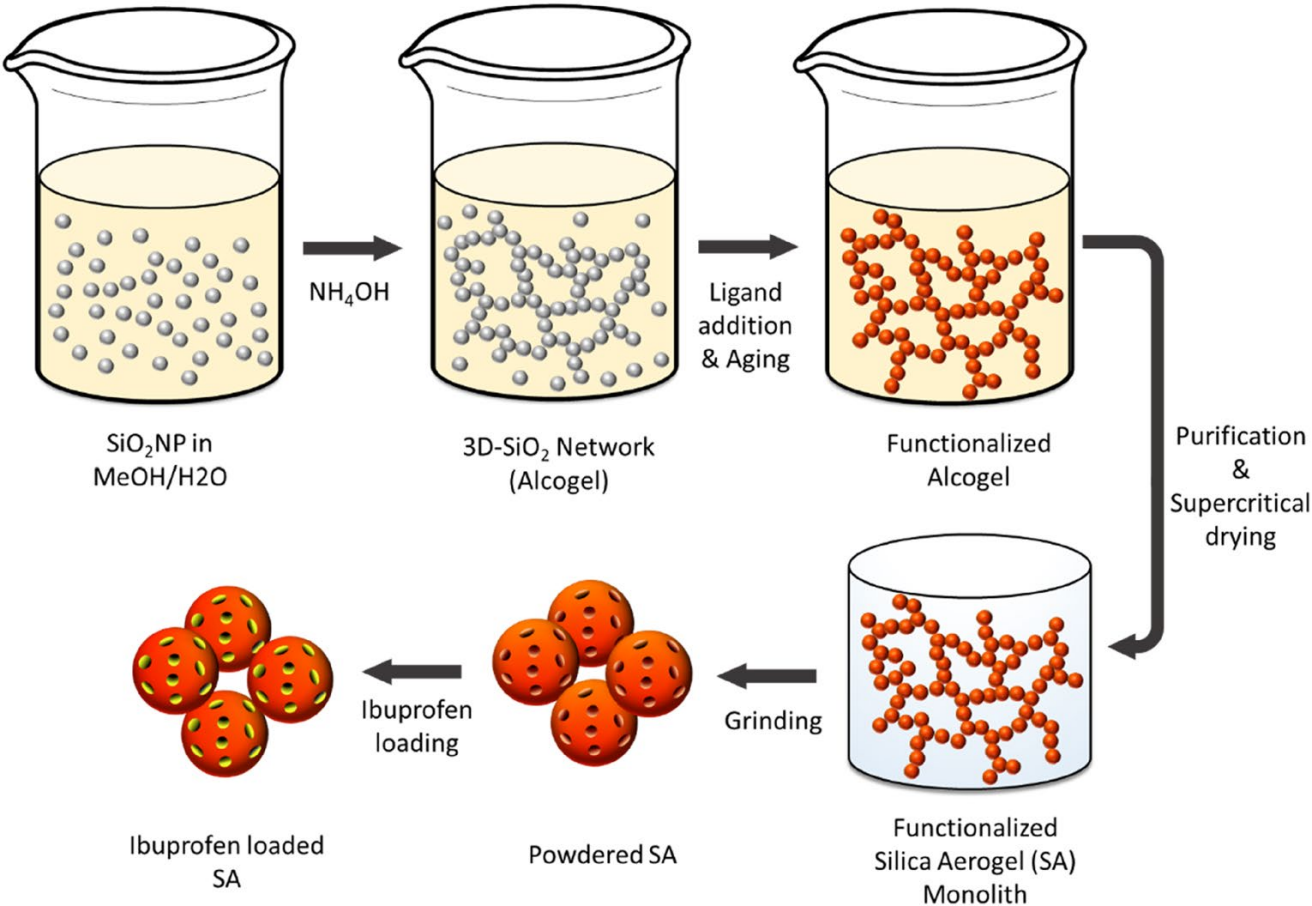
Schematic depiction of the SA multi-step synthesis of ibuprofen loaded SA.

Herein, we present a profound study on process-related and physico-chemical determinants for processing long-term stable ibuprofen-SA formulations (Figure 1). To this end, the influence of macrostructural inhomogeneities of monolithic SA on the microstructural integrity was elucidated. Moreover, powdering of SA monoliths was comprehensively investigated with regard to the preservation of the outstanding microstructural SA properties which are decisive for an effective drug loading of SA. Textural characteristics were determined via nitrogen sorption analysis, small-angle X-ray scattering (SAXS), scanning electron microscopy (SEM) and dynamic image analysis (QICPIC). In addition to an optimized process engineering, chemical properties of the matrix were selectively tuned: by means of surface functionalization with (3-aminopropyl)triethoxysilane (APTES) and trimethylchlorosilane (TMCS), specific drug-carrier interfaces were designed for improved drug adsorption and defined drug release behaviors. Subsequently, post-synthetic drug loading via co-milling and the melting method was systematically examined. Therefore, formulation and process parameters were studied in order to assess their influence on product properties and to improve process performance as well as the final product properties. Drug loading was verified via dynamic scanning calorimetry (DSC). Finally, the drug release kinetics of co-milled and melted ibuprofen-SA formulations with different surface properties were determined based on UV-VIS analyses. The processed SA revealed highly advantageous properties, combining excellent drug loading capacities, tailorable release kinetics and remarkable long-term drug stability, proving a great potential of the SA as a versatile drug carrier platform technology.

## Material and methods

### Chemicals and reagents

Ammonium hydroxide solution (28–30%), (3-aminopropyl)-triethoxysilane (APTES, 99%), trimethylchlorosilane (TMCS, ≥ 98%), ethanol (99.8%), hydrochloric acid (HCl, 37%), methanol (MeOH, ≥ 99.8%), ninhydrin (≥ 99%) and tetramethyl orthosilicate (TMOS, ≥ 98%) were obtained from Merck KGaA (Darmstadt, Germany). Carbon dioxide (technical grade) was purchased from Westfalen AG (Münster, Germany). Ibuprofen was kindly gifted from Euro OTC Pharma GmbH (Bönen, Germany).

Unless otherwise specified, ultrapure water was used.

## Methods

### Synthesis

SA were synthesized by sol-gel polymerization of TMOS according to Alnaief & Smirnova (2010) with slight modifications. First, TMOS was mixed with MeOH and water with a molar ratio of 1 mol_TMOS_:2.4 mol_MeOH_:1.3 mol_H2O_. Since TMOS possesses a relatively high rate of hydrolysis, no HCl was used as catalyst (Innocenzi, 2019). The prepared sol was stirred at 670 rpm for 30 min at room temperature. Then, additional water and ammonia solution (28–30%) were added to obtain the final molar ratio of 1 mol_TMOS_:2.4 mol_MeOH_:4 mol_dH2O_:0.01 mol_NH4OH_. The mixture was stirred for one additional minute and subsequently transferred to a cylindrical vessel and sealed with parafilm to prevent solvent evaporation. The alcogel was formed after 2 min and then aged in 25 mL MeOH for 7 days with a solvent exchange step after 24 h. Thereafter, the alcogels were supercritically dried using a SPI-DRY E3100 critical point dryer (CPD) from Quorum Technologies (West Sussex, United Kingdom). To this end, the pressure chamber was filled with MeOH and loaded with the aged alcogels. Afterwards, the CPD was hourly flushed for 15 min with liquid CO_2_ over a span of 4 h, followed by a 20 h impregnation time and a final analogous flushing step to ensure that MeOH is thoroughly removed from the internal gel network. Consequently, liquid CO_2_ was rendered supercritical by raising the temperature to 35 °C resulting in a concomitant pressure increase to approximately 90 bar. The supercritical state was maintained for one hour and the system subsequently depressurized in a controlled manner to atmospheric pressure over 90 min (1 bar min^−1^) to minimize pore collapse and yield the highly porous and homogenous monolithic aerogels.

### Process-related structural defects of the SA

**Figure 2.**
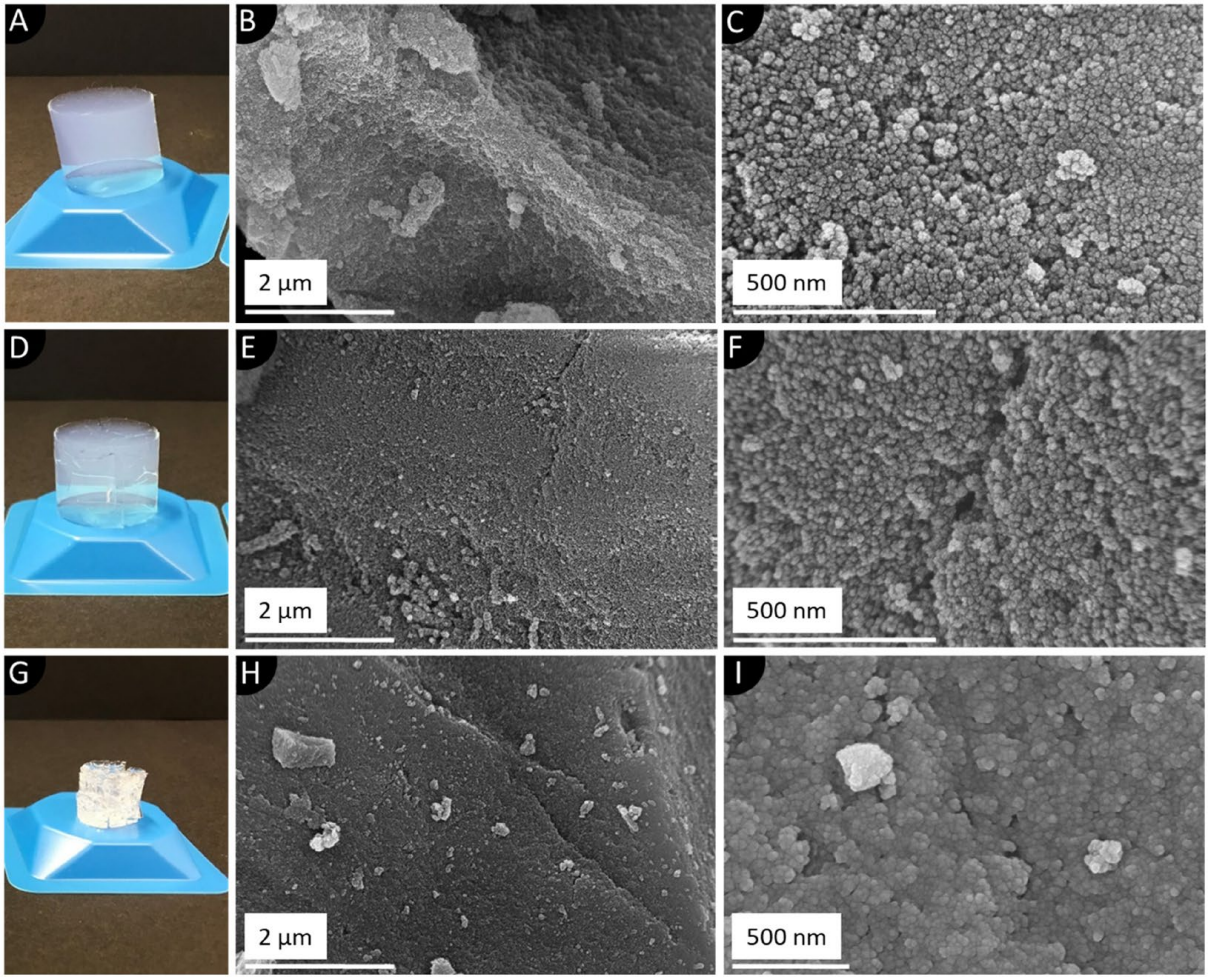
Dried monolithic SA with different degrees of process-related structural defects: Photographs and SEM images of an intact (A-C), cracked (D-F) and collapsed (G-I) SA. The collapse of the latter was induced by drying the alcogel under ambient atmosphere.

Since the monolithic structure of the aerogels can experience structural deformations during the synthesis and drying of the SA, the influence of macrostructural inhomogeneities on microstructural characteristics was investigated. First, two different batches of SA were synthesized under idential conditions as described above. However, the second batch (Figure 2D) was depressurized in an accelerated manner with a rate of 2 bar min^−1^. Furthermore, a third sample was synthesized which was, however, dried under ambient conditions in an oven at 80 °C for 24 h in order to facilitate a thoroughly collapsed macrostructure (Figure 2G). All dried samples were finally powdered using the vibratory ball mill (VBM; CryoMill, Retsch, Haan, Germany) for 15 min at ambient temperature and 15 Hz.

### Grinding studies

In order to determine a reproducible procedure to obtain homogenously powdered samples, monolithic SA were ground using different comminution methods. More specifically, pre-grinding was performed using a mortar and pestle for 3 min. The pulverized samples were then subsequently classified by a sieve shaker (AS 200, Retsch, Haan, Germany) with a mesh size of 125 μm for 1 min. Grinding and sieving of the coarse-grained, separated particles was repeated for three times to minimize material loss. In addition, SA were ground using a vibratory ball mill. For this purpose, a stainless steel cryo-mill chamber of 50 mL capacity was filled with six stainless steel grinding media of 10 mm diameter. For a defined milling procedure, the influence of the milling time and frequency on the microstructure of the aerogels were varied, as specified in Table S1. Furthermore, a comparison between grinding at ambient temperature and under cryogenic conditions (−196 °C) was carried out (CVBM). The system was cooled down throughout the whole procedure by attaching an auto-filling liquid nitrogen dewar (Retsch, Haan, Germany) to the CryoMill. Before any milling was commenced, a precooling step was applied. That entailed the mill operating at a vibrational frequency of 5 Hz while liquid nitrogen circulated around the stainless-steel chamber jacket until a temperature of −196 °C was reached. Afterwards, the samples were milled at different frequencies and times as listed in Table S1.

**Table 1.** Grafting density (δ_Ligand_), specific surface area (S_A_), pore volume (V_P_), pore size (d_P_) and the C value of the BET equation of plain and functionalized SA.

Sample	N%	C%	δ_Ligand_/molecules nm^−2^	S_A_/m² g^−1^	V_P_/cm³ g^−1^	d_P_/nm	C
SA	0	4.8 ± 0.64	–	976 ± 29	2.6 ± 0.02	11.8 ± 0.87	92 ± 6
SA@APTES	3.0 ± 0.10	–	1.68 ± 0.05	766 ± 45	2.5 ± 0.13	12.9 ± 0.61	53 ± 3
SA@TMCS	–	7.5 ± 0.12	0.54 ± 0.02	839 ± 51	2.8 ± 0.14	13.4 ± 0.73	34 ± 4

APTES (SA@APTES) and TMCS (SA@TMCS) graftings were carried out for 24 h and 48 h, using a molar ratio of 0.5 mol_Ligand_ per mol_TMOS_. While the determination of the grafting density of APTES was calculated based on the recorded nitrogen content (N%), the amount of TMCS per nm^2^ surface was derived from the measured carbon content (C%). Each value is based on three independent samples.

### Surface functionalization

For the preparation of SA with tailored surface chemistry, the primal hydroxyl groups on the SA surface were modified using APTES or TMCS resulting in hydrophilic and hydrophobic SA, respectively. The effect of different parameters on the silanization processes were investigated by varying the added amount of APTES and TMCS from 0.01 to 1.0 mol in relation to the molar amount of TMOS. Secondly, the influence of the silanization time on the resulting grafting densities was examined. To this end, alcogels were prepared as described before and subsequently aged for 24 h in MeOH. Afterwards, the respective functionalization ligand was added dropwise via a syringe at room temperature. The samples were then placed on a shaker (VXR basic Vibrax, IKA, Staufen im Breisgau, Germany) at 100 rpm for different time intervals. The subsequent removal of unreacted ligand molecules was carried out over two days by performing 3 washing steps per day at 2 h intervals. To prove the complete removal of unreacted APTES molecules, the ninhydrin test was applied (Kockmann et al., 2015), revealing a negative result. After purification, the alcogels were dried in an oven at 80 °C for 20 h.

### Drug loading by co-milling of the prepared formulations

Ibuprofen loading of SA was carried out using supercritically dried and functionalized SA. Therefore, alcogels were functionalized with 0.5 mol_ligand_/mol_TMOS_ for 48 h and finally dried under supercritical conditions as described before. No additional washing steps were necessary, since remaining unbound functionalization agent was washed out by the CO_2_-flushing processes. Functionalized and dried SA were then ground to fine powder using the VBM for 15 min at 15 Hz and at room temperature. Subsequently, ibuprofen was additionally charged into the same chamber of the VBM in defined ratios such that all prepared samples contained 1 g of physical blend. Analogous to the grinding studies, six stainless steel grinding balls (10 mm in diameter) were used for the drug loading process. Co-milling experiments were performed for 15 min at 15 Hz and at room temperature. In addition, two milling control tests were conducted: first, 1 g of plain ibuprofen was milled under the same conditions in order to determine the influence of the bare milling process on its structural properties. Secondly, powdered SA was milled for a second time under the same conditions but without the addition of ibuprofen in order to determine the influence of the milling process on the SA microstructure.

### Drug loading by melting of the prepared formulations

Loading of ibuprofen into the powdered (functionalized and non-functionalized) SA was alternatively conducted by the melting method. A physical mixture of ground SA and ibuprofen powder was heated above the melting temperature of ibuprofen (75 °C) in a furnace at 100 °C for 24 h. Drug loading efficiency was studied as a function of different drug-carrier ratios, as well as of different interfacial properties (hydroxyl-, amino- and methyl terminated surface groups). The total mass of all examined formulations was kept constant at 1 g.

### In vitro dissolution studies

Dissolution studies were conducted following the recommendations of the International Pharmaceutical Federation (paddle test at sink conditions) (Siewert, 1996). Therefore, the USP apparatus II was utilized with 900 mL of 0.1 M HCl (gastric pH of 1.2) as dissolution medium. The stirring speed was set to 100 rpm and temperature to 37 °C. The amount of each ibuprofen-SA formulated sample was varied in order to assure that the final ibuprofen content was equal to approximately 10% of its maximum solubility in 0.1 M HCl (sink conditions). Hence, 10.8 mg of ibuprofen were used throughout all dissolution studies. At defined time intervals, 2 mL samples were withdrawn, immediately filtered through a 0.45 μm Nylon filter and always replaced with fresh dissolution medium. Subsequently, the dissolved ibuprofen content was determined via UV/VIS-spectroscopy at a wavelength of 220 nm using a Specord 210 Plus (Analytik Jena GmbH, Jena, Germany). Finally, the relative amount of ibuprofen was calculated with respect to the initially utilized 10.8 mg per sample.

## Characterization

The specific surface area *S*_A_, pore size diameter d_pores_ and pore volume V_p_ were determined by nitrogen sorption analysis using the surface area and porosimetry analyzer ASAP 2460 from Micromeritics (Norcross, GA, USA). Prior to the gas sorption measurements, approximately 100 mg of each powdered sample was degassed under vacuum for 2 h at 200 °C to remove residual solvent and moisture within the microstructure of the aerogels. The specific surface area S_A_ was determined within a relative pressure range p/p_0_ between 0.05 and 0.3 of the adsorption isotherm using the multipoint Brunauer, Emmett, and Teller (BET) analytical method (Brunauer et al., 1938). Pore size distribution and pore volume were obtained by applying the Barrett, Joyner, and Halenda (BJH) method to the recorded desorption isotherms (Barrett et al., 1951).

Particle morphology of the aerogel samples was studied by scanning electron microscopy (SEM) on a Helios G4 CX (FEI Deutschland GmbH, Frankfurt am Main, Germany). The powdered samples were prepared on a double-sided adhesive carbon pad and sputter-coated with a platinum layer of 4 nm. Particle sizes of the nanostructured SA network were determined by measuring 50 SiO_2_-nanoparticles, using the ImageJ software (Version 1.42q).

The particle size distributions with the respective x_50_ median (d_particles_) of the aerogel powder were determined by an image analysis system that comprises a dynamic image sensor (QICPIC; Sympatec GmbH, Clausthal-Zellerfeld, Germany), a gravity disperser GRADIS/L and a dosing unit VIBRI/L. In order to ensure an optimum concentration of particles within the measuring zone, an initial vibration intensity of 10% was chosen and the measurement time was set to 300 s. Analysis was carried out with at least 100,000 particles in triplicates.

Quantitative examinations of the degree of functionalization were performed by elemental analysis using a FlashEA 1112 from Thermo Fisher Scientific (Waltham, MA, USA). The molar concentration (c) of chemisorbed APTES ligand per g_SA_ was determined from the ratio of the measured nitrogen weight content (x%) to the molar mass (Zarinwall et al., 2021c):

(1a) $$c_{\mathrm{APTES}}=\frac{\left(({x\%}_{\mathrm{mod}}-{x\%}_{\mathrm{unmod}})/100\right)}{14\;g*mol^{-1}}\text{ given in }mol_{\mathrm{APTES}}*g_{SA}^{-1}$$

Likewise, the molar concentration of bound TMCS was determined from the ratio of the measured carbon weight content to the molar mass. Since one TMCS molecule possesses three methyl groups, a total molar mass of 36 g/mol needs to be considered for the calculation of the TMCS concentration.

(1b) $$c_{\mathrm{TMCS}}=\frac{\left(({x\%}_{\mathrm{mod}}-{x\%}_{\mathrm{unmod}})/100\right)}{36\;g*mol^{-1}}\text{ given in }mol_{\mathrm{TMCS}}*g_{SA}^{-1}$$

The number of molecules grafted on the SA surface per square nanometer (referred to as grafting density δ_ligand_) was then calculated as:

(2) $$\delta_{\mathrm{ligand}}=\frac{c*N_{A}}{S_{A}*10^{18}}\text{ given in }\mathrm{molecules}*nm^{-2}$$

where N_A_ is the Avogadro number (6.02 ⋅ 10^23^ mol^−1^) and S_A_ the specific surface area in m^2^ g_SA_^−1^.

To elucidate the coordination mode of the ligands, thermogravimetric analysis (TGA; STA7200, Hitachi High-Tech Analytical Science, Oxford, UK) was linked with mass spectrometry (GSD 320 Thermostar TM, Pfeiffer-Vacuum, Asslar, Germany) via a coupling device (280 °C, REDshift, San Giorgio in Bosco, Italy). 10–12 mg of the samples were measured in alumina crucibles at a heating rate of 10 K/min in a nitrogen flow of about 200 mL/min up to 600 °C. The flow was redirected into a heated transfer capillary (deactivated fused silica tubing; inner diameter: 0.15 mm; outer diameter: 0.22 mm; kept at 280 °C; split of 1:200) and the coupling into the inlet (150 °C) of the mass spectrometer. The mass spectrometer was operated in the electron ionization mode at 70 eV with a secondary electron multiplier at 1080 V as detector. Measurements were performed in multiple-ion detection mode (MID) with 200 ms measurement time. The measured ion currents *I(T)* were baseline-corrected to their minimum *I_min_* and normalized to the weight of the sample *m_sample_* to obtain comparable ion currents *I_rel_(T)* according to:

(3) $$I_{\mathrm{rel}}(T)=\frac{I(T)-I_{\min}}{m_{\mathrm{sample}}}$$

Thermal analyses were further performed by dynamic differential scanning calorimetry (DSC). Therefore, the powdered samples were loaded in an aluminum crucible with a perforated lid and subjected to a heating/cooling profile (20 °C to 100 °C) in a DSC apparatus (DSC 3^+^, Mettler Toledo GmbH, Columbus, Ohio, USA) with a heating rate of 10 °C/min under a nitrogen purge gas flow. Based on the so determined enthalpy of fusion of plain (H_ibu_) and embedded ibuprofen (H_sample_), the content of amorphized (A%) ibuprofen within the formulation was calculated by further taking into account the respective mass fraction of ibuprofen (ω_ibu_) (Patel et al., 2014):

(4) $$A\%=(1-\frac{H_{\mathrm{sample}}}{H_{\mathrm{ibu}}\text{ * }\omega_{\mathrm{ibu}}})*100\text{ given in \%}$$

SAXS measurements were performed with a SAXSess mc^2^ system from Anton Paar (Graz, Austria) using a Cu-*K*α radiation source (wavelength: 0.154 nm, current: 40 kV, voltage: 50 mA) and a constantly cooled PI-SCX:4300 CCD detector (T = −40 °C). At room temperature, a 1 mm flow-through quartz cuvette was positioned within the slit collimated beam path at a sample-to-detector distance of 309 mm. Exposure times were between 0.1 and 100 s and the measuring times between 2 and 1000s. The scattering data were routinely corrected (Pauw et al., 2017) with respect to instrumental background noise, transmission and slit-smearing effects. Basic data processing was conducted in SAXSquant (Anton Paar GmbH, Austria), whereas desmearing and the indirect fourier transformation (IFT) were performed using generalized indirect Fourier transformation (GIFT) from the PCG software package (Bergmann et al., 2000). The IFT translates the scattered data into real space resulting in a model-free pair distance distribution function (P(*r*)) that visualizes all frequencies of lengths between individual scatters within the system. The relatively most occurring distance is observable by the mode of the P(*r*), which equals the radius (R_max_) for spherical particles.

## Results and discussion

### Investigation of SA structure and its change during processing

**Figure 3.**
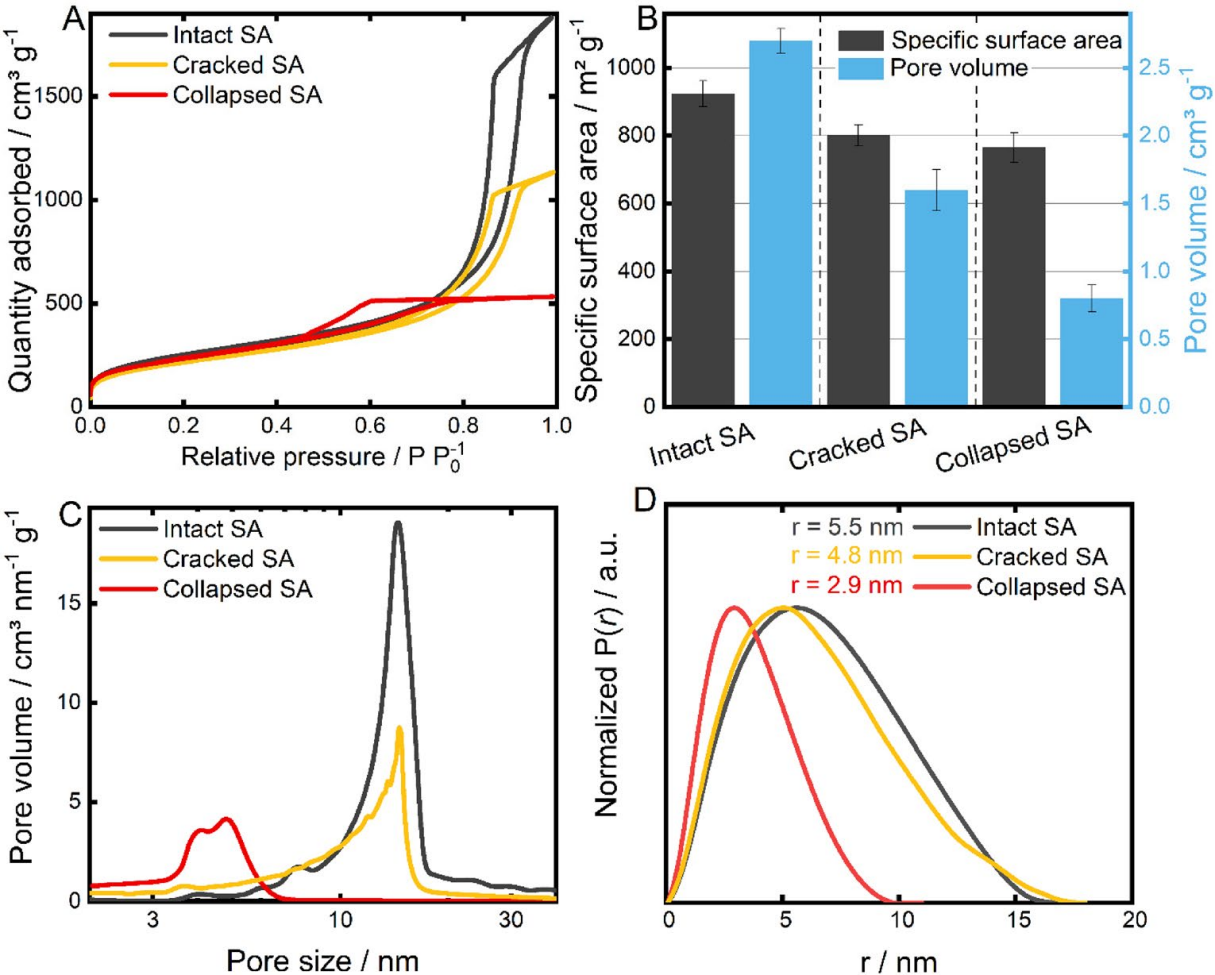
A) Nitrogen sorption isotherms, B) specific surface area as well as pore volume, and C) pore size distributions determined from the respective adsorption and desorption branches, respectively. E) Normalized pair distance distribution functions P(*r*) with the respective R_max_ values.

The macroscopic structure of the synthesized SA is decisively shaped by the degree of homogeneity of the manufactured monoliths after the final drying step (Figure 2A, D, G). These forms of structural defects can emerge throughout the synthesis process as a result of system-related uncertainties even though a controlled supercritical drying step is applied (Job et al., 2005). Hence, the effect of monolithic cracking during the drying process was investigated. Although not the monolithic structure but rather the powdered SA is intended to be utilized for the targeted application, it is of great importance to determine the influence of macroscopic structural defects on the inner micro-textural properties for the establishment of a defined manufacturing procedure. Hence, the morphology of intact, cracked and collapsed SA monoliths was studied by SEM analysis. Micrographs of highly homogenous SA monoliths reveal a rough and sponge-like microstructure (Figure 2B). Upon greater magnification, a nanostructured, three-dimensional network of approx. 25 nm-sized spherical silica particles can be observed (Figure 2C). Moreover, interparticulate hollow spaces in the network were successfully resolved too. In case of monoliths with modest structural deficits, a slightly smoothed surface can be seen (Figure 2E) with, however, a similar average arrangement of the open-pore structured silica network (Figure 2F). In contrast, monoliths dried under ambient pressure exhibit not only a much smoother surface (Figure 2H), but also a highly densified particle arrangement. This can be related to intense capillary stress exerted on the pores, resulting in a collapsed and densified network (Figure 2I). To get a more profound understanding of the altered microstructural properties, quantitative information was obtained by means of nitrogen sorption analyses and SAXS measurements (Figure 3).

The recorded nitrogen adsorption-desorption isotherms (Figure 3A) of all analyzed samples can be classified as type IV isotherms that are known to arise from materials with a micro- and mesoporous structure (Bardestani et al., 2019). As depicted in Figure 3A, the respective desorption branches exhibit hysteresis loops which are generally attributed to the capillary condensation occurring in the mesopores (Liu et al., 2009). However, two distinct types of hysteresis curves are evident: While intact and cracked SA show type H1 hysteresis loops indicating the presence of cylindrical pores, the collapsed SA exhibits type H2 associated with bottle-neck-shaped pores (Bardestani et al., 2019). Quantification of the physical attributes was performed by applying the BET and the BJH evaluation models on the adsorption or desorption branch, respectively, in order to determine the corresponding specific surface area and the related pore volume of each prepared SA sample (Figure 3B). Accordingly, the intact and crack-free synthesized SA shows the highest obtained specific surface area (977 ± 29 m^2^/g) followed by the SA with a cracked (802 ± 31 m^2^/g) and collapsed (765 ± 44 m^2^/g) macrostructure. In addition, a remarkable loss of pore volume by 38% and 70% was determined for cracked and collapsed SA in comparison to the unscathed sample. In correspondence, a reduction of the average pore size from 11.3 nm to 8.8 nm and 5.0 nm was determined (Figure 3C). This was successfully confirmed by SAXS measurements (Figure 3D). The SAXS-derived diameters (calculated via R_max_ values) amount to 11.0 nm (intact SA), 9.6 nm (cracked SA) and 5.8 nm (collapsed SA). The slight deviation can be attributed to differences in the measurement principle: While the recordings of nitrogen desorption isotherms serve for the BJH evaluation method and thus for the determination of the presented pore size distribution, SAXS measurements are based on elastic scattering of X-rays passing through a sample. According to the literature, the texture of SA can be described by a ‘two-density’ model consisting of the electronic density of the three-dimensional matrix and that of the pores (Craievich et al., 1986). Hence, an evidenced and differentiated evaluation of the SAXS measurements necessitates further complementary analysis data for an unambiguous assignment of the recorded scattering data: SEM study revealed an approximate particle size of the quasi-spherical SA clusters of 25 nm (Figure 2C). Furthermore, the recorded nitrogen isotherms (Figure 3A) pointed out the presence of cylindrical pores for SA with no or only modest macroscopic defects. SAXS measurements also indicate a cylindrical morphology on the basis of the obtained and normalized pair distance distribution functions (Glatter, 1979). However, no signs of structural changes of the pores were recorded by SAXS measurements for collapsed SA. Nonetheless, the determined nanostructural features can be correlated with the presence of pores within the SA samples, which complements the findings based on nitrogen sorption and SEM analysis. The registered microstructural changes, i.e. loss of pore volume, reduction in pore size and presumably a change in pore morphology, can be attributed to a moderate or stronger collapse of pores or shrinkage of the gel network as a result of acting capillary forces. Since regular and accessible open pores are decisive for a high drug loading efficiency, these results hence demonstrate the necessity of a controlled manufacturing process with slow depressurization correlating with a high product quality.

### Grinding studies

**Figure 4.**
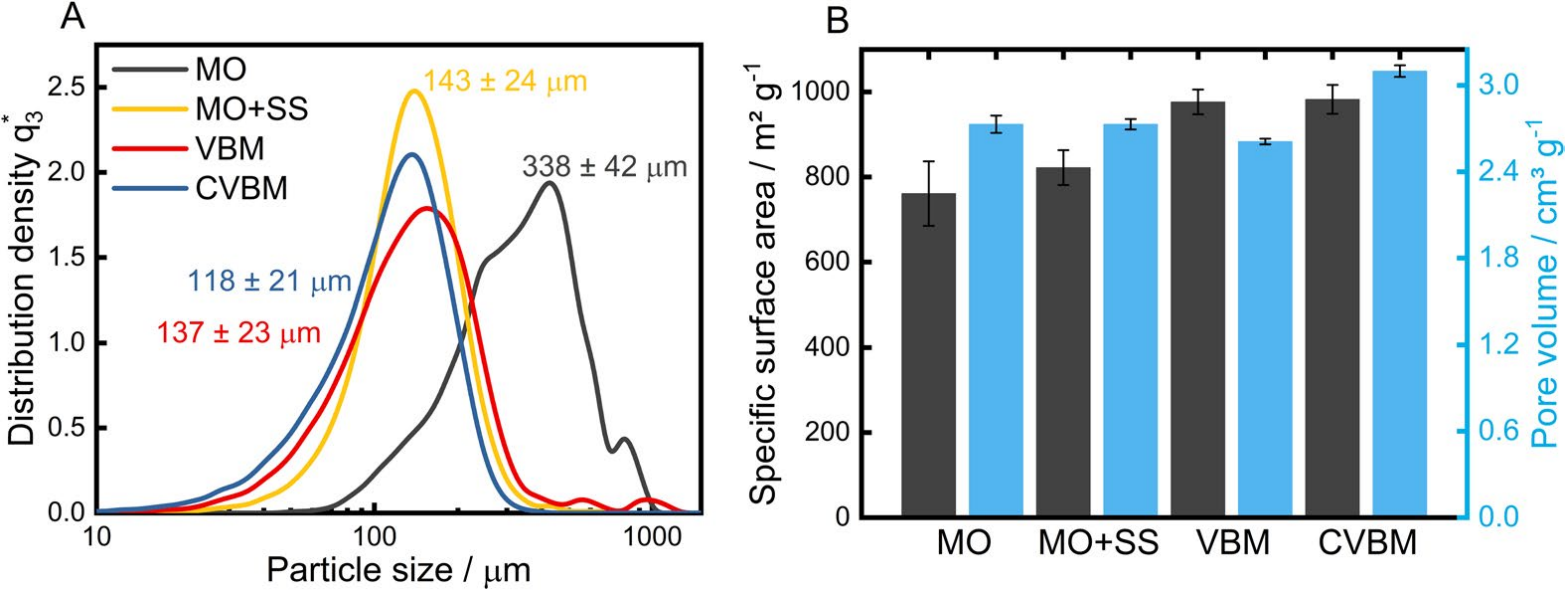
A) Density-based particle size distributions including the respective X_50_ value after grinding of SA using a mortar and pestle (MO), applying subsequent classification using a sieve shaker (MO+SS) and powdering by a vibratory ball mill at room temperature (VBM; 15 min, 15 Hz) and under cryogenic conditions (CVBM; 15 min, 15 Hz). Measurements were performed in triplicates. B) Resulting specific surface areas and pore volumes after each method of SA comminution. Each bar represents the mean ± SD of at least 3 independent experiments.

Systematic analysis of the post-synthetic drug loading of SA requires a prior comminution of the monolithic structure into a homogenous powder in a defined and reproducible manner for an improved material handling. However, as shown before, macroscopically induced structural changes exert an influence on the microstructural properties of the SA. Hence, the grinding process of the monolithic SA was thoroughly examined in order to achieve homogenously powdered SA while retaining the highly porous microstructure. Therefore, different grinding methods were used and the products consequently characterized by image analysis-based particle size measurement (QICPIC) and nitrogen sorption analysis (Figure 4). The resulting particle size distributions are reported in Figure 4A. SA crushed in a mortar (MO) exhibits the broadest particle size distribution with a mean diameter of 338 ± 42 μm and a slight bimodal tendency. Fractions obtained by sieving (SS) and milling (VBM and CVBM) display a narrower size distribution with a mean diameter of 143 ± 24 μm, 137 ± 23 and 118 ± 21 μm, respectively. These differences in powder homogeneity were confirmed by SEM images of the comminuted samples (Supplementary Information, Figure S1). Furthermore, the specific surface area and the related pore volume of the prepared SA samples were studied by nitrogen sorption analysis (Figure 4B). The lowest obtained specific surface area amounts to 761 ± 76 m^2^/g, which was greatly enhanced by the use of a better suited comminution method. In comparison, introducing a subsequent classification by the means of a sieving procedure substantially narrows the recorded standard deviation by 73% and thus leads to higher specific surface area of 802 ± 21 m^2^/g. A further increase of the specific surface area was obtained using the VBM under ambient (976.7 ± 29 m^2^/g) and cryogenic (982.5 ± 34 m^2^/g) conditions, presumably due to additional break-up of particle agglomerates that leads to a better accessibility of the inner SA surface for the nitrogen analyte. Thus, the comminution of the SA monoliths does not entail any detrimental deformation of the porous SA structure. Nevertheless, it needs to be stated that the resulting structural properties are significantly dependent on the utilized milling process parameters, such as grinding time, oscillation frequency and temperature (Supplementary Information, Tables S1 and S2).

### Surface functionalization

**Figure 5.**
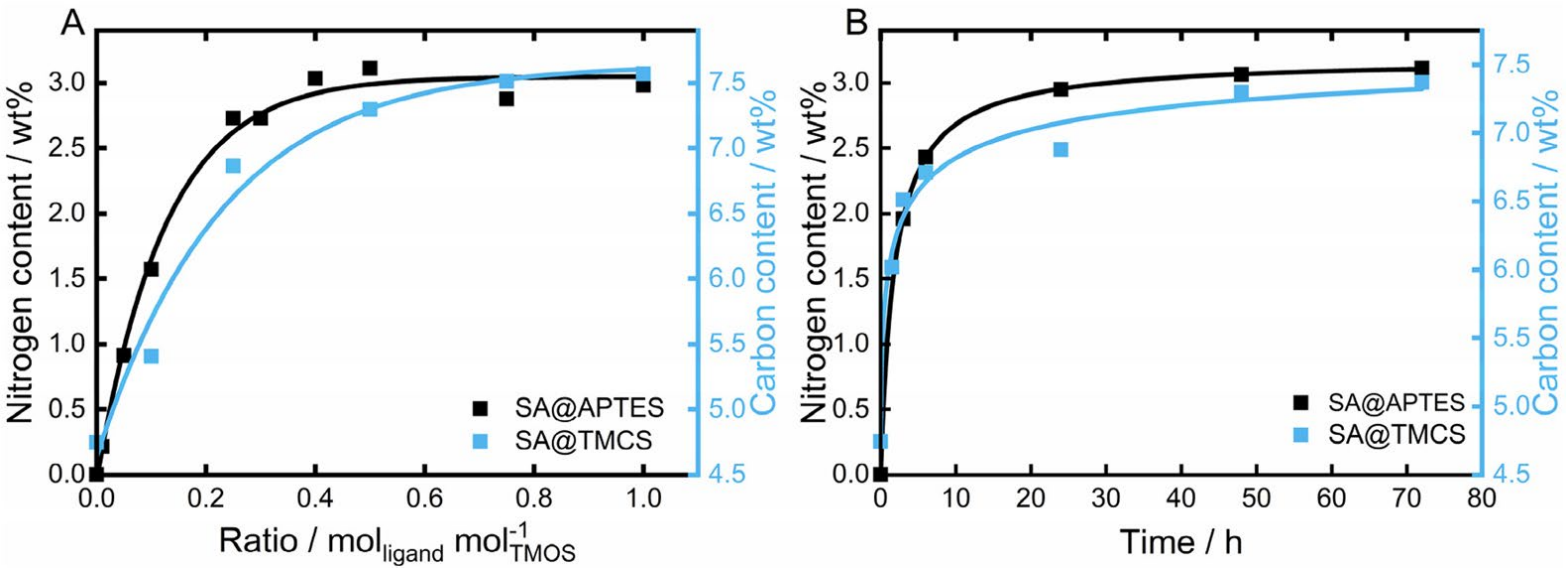
Elemental analysis of functionalized SA with respective nitrogen and carbon content as a function of A) the initially added molar amount of the respective ligand and B) of the reaction time.

**Figure 6.**
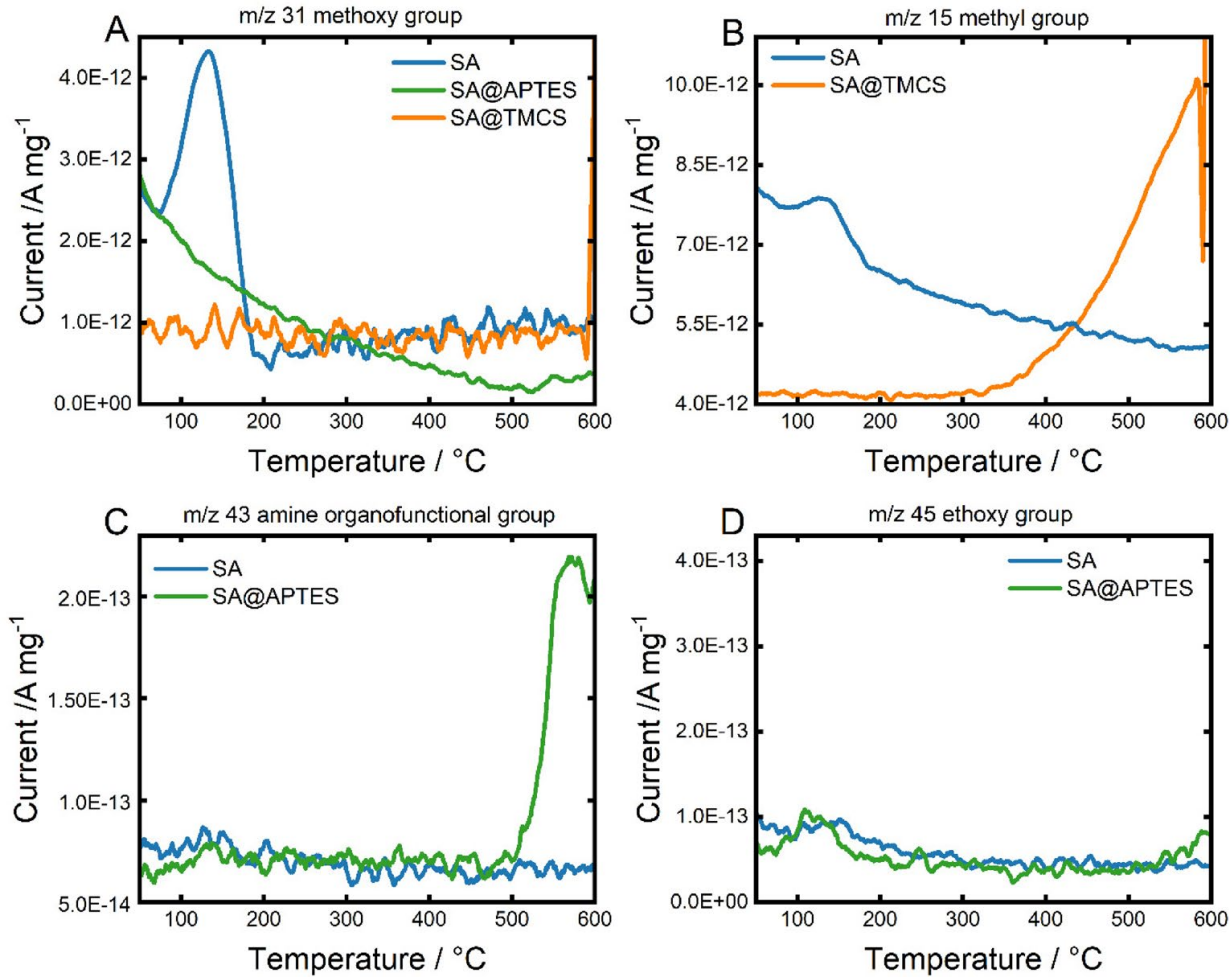
Temperature-resolved signals of A) *m/z* 31 (methoxy group), B) *m/z* 15 (methyl group), C) *m/z* 43 (amine group) and D) *m/z* 45 (ethoxy group). Both analyzed samples, SA@APTES and SA@TMCS, were initially modified with a molar ratio of 0.5 mol_ligand_/mol_TMOS_.

Since the nature of the drug-matrix interface is decisive to the drug loading efficiency and release (Veres et al., 2015), a comprehensive characterization of the functionalization process was conducted prior to the drug loading procedures. SA was modified with APTES and TMCS in order to achieve versatile surface chemistry and thus specifically adjust the affinity of the SA matrix to ibuprofen as well as to the surrounding media after an oral application of the formulation. As the type of drying (ambient or supercritical atmosphere) did not show any influence on the grafting process (Supplementary Information, Figure S2), the study of the parameter dependence was carried out with gels dried under ambient atmosphere for a higher throughput. The resulting degree of functionalization was examined by elemental analysis (Figure 5) since both ligands introduce characteristic functional entities, i.e. amino-(APTES) and methyl groups (TMCS). Consequently, the determined nitrogen and carbon content was plotted as a function of the added molar amount of APTES or TMCS with respect to the molar amount of TMOS used initially for the SA synthesis (Figure 5A). Analysis of bare SA (shown at the origin of both graphs) revealed that no nitrogen was present in the reference sample. In contrast, the detected carbon content amounts to 4.8 wt%, originating from an incomplete hydrolysis of TMOS (see below). The nitrogen and carbon content of modified SA strongly increases with the initially used ligand content indicating a quantitative coordination of the ligands onto the SA surface. A further increase in the added amount of the ligand results in a plateau at 0.4 mol_APTES_/mol_TMOS_ and 0.5 mol_TMCS_/mol_TMOS_. In addition, the temporal dependency of both grafting processes was examined (Figure 5B). Accordingly, reaction times of approximately 24 h for APTES and 48 h for TMCS are required to achieve a complete surface coverage since no further increase in the nitrogen and carbon content was observed for longer functionalization times. The obtained converging behavior indicates a complete occupation of all accessible binding sites under formation of a homogenous monolayer (Zarinwall et al., 2021; 2021b) which is important for achieving the highest possible surface coverage. To specifically elucidate and evidence the nature of coordination, e.g. the presence of individual silane patches on the surface vs. the formation of a mono- or polylayer, TGA-MS analyses were performed (Figure 6).

The MID mass spectra of unmodified SA reveal the presence of a *m/z* 31 fragment (Figure 6A) that can be ascribed to the thermal decomposition of methoxy groups (CH_3_O^+^) remaining within the colloidal SA network due to partially incomplete hydrolysis of the TMOS precursor. In contrast, SA@APTES and SA@TMCS do not exhibit an analogous signal indicating an accompanied substitution of the methoxy group throughout the functionalization process. The successful grafting of TMCS can be deduced from the distinct signal in Figure 6B (*m/z* 15, CH^+^_3_) evolving at 350 °C which represents a characteristic temperature range for the thermal decompositon of bound TMCS (Rao et al., 2007). With respect to the APTES functionalization, the most significant signal was detectable for *m/z* 43 which can be attributed to a H_2_N-CH = CH^+^_2_ or H_3_C-CH_2_-CH^+^_2_ fragment representing the aminopropyl moiety (Figure 6C). According to our previous study (Zarinwall et al., 2021), the high thermal stability of the functional group against decomposition in addition to the absence of a signal for the ethoxy group of non-hydrolyzed APTES at *m/z* 45 (Figure 6D) indicates an intermolecularly cross-linked and covalently bound APTES monolayer on the SA surface.

The corresponding grafting densites for APTES and TMCS can be calculated using Eqs. (1a, b) and (2). Accordingly, up to 1.68 ± 0.05 molecules_APTES_/nm^2^ and 0.54 ± 0.02 molecules_TMCS_/nm^2^ were attached to the SA matrix (Table 1). The difference can be attributed to increased molecular volume thus to enhanced steric hindrance by the trimethyl group of TMCS compared to the aminopropyl moiety of APTES (Marcinko & Fadeev, 2004; Pujari et al., 2014). Moreover, an intriguing question arises about possible textural changes in the gel network as a consequence of possible further condensation reactions after the addition of the surface modifiers. Since functionalization was performed via co-condensation subsequent to the complete alcogel formation, it can be assumed that the ligands primarily condense on the outer and inner surface of the SA particle clusters and do not cause any major structural rearrangements. This was successfully confirmed by nitrogen sorption analyses of all prepared samples (Table 1). Whilst modest changes were observed for the specific surface area of APTES- and TMCS-modified SA, no significant differences in the pore volume and pore diameter were determined in comparison to the unmodified sample. Hence, it is likely that primarily some accessible binding sites on the SA surface were occupied, leading to a reduced adsorption capacity of nitrogen during the sorption analysis and consequently to a lowered specific surface area. A simplistic qualitative approach for proving altered accessible binding sites is achieved by assessment of the C constant of the BET equation: the lower the C constant value, the lower the affinity of the nitrogen molecules toward the surface of the adsorbent and thus its hydrophilicity (Sing, 1998). Plain SA presented values of C in the order of 92 ± 6. In contrast, C values measured for APTES-functionalized SA were reduced to 53 ± 2. Hüsing *et al.* proposed that a decrease of the C value for APTES-functionalized surfaces may be attributed to the formation of intra- and intermolecular hydrogen bonds of the amino moiety with the nitrogen molecules therefore adsorbing on the non-polar propylene spacer (Hüsing et al., 1999). Nonetheless, since TMCS solely possesses non-polar terminal methyl groups, a further decrease of the C value (34 ± 4) followed and thus confirmed the differed available binding sites on the SA surface, which validates the made assumption of an occupation of the terminal SA binding sites rather than an altered network structure.

### Drug loading

**Figure 7.**
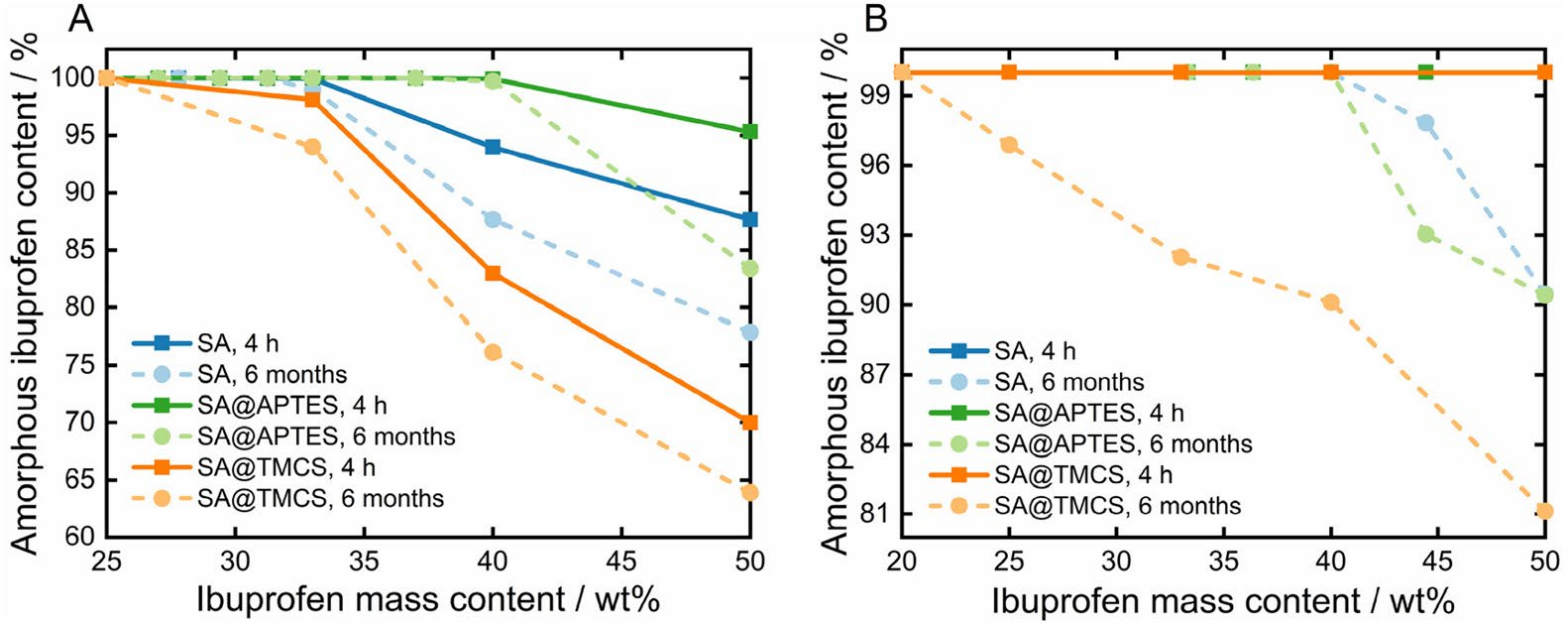
Resulting amorphous ibuprofen content as a function of its concentration for differently functionalized SA after A) co-milling and B) melting of the physical blends.

Co-milling and melting of physical blends of tailored or plain SA and ibuprofen was conducted in order to assess the process-related drug loading efficiency as well as the maximum amorphization capacity of the respective SA matrices. To this end, the phase composition of ibuprofen was analyzed and quantified via DSC studies (Supplementary Information, Figures S3 and S4). Accordingly, unprocessed ibuprofen exhibits a single endothermic melting peak which verifies its crystalline structure. The thereof derived enthalpy of fusion (−124.6 J/g) allows to ascertain the content of amorphized ibuprofen within the prepared formulations using Eq. (4). Differently functionalized SA were first co-milled with various mass fractions of ibuprofen (Figure 7A) resulting in a significant reduction of the DSC peak area or even the complete absence of the endothermic signal due to amorphization (Supplementary Information, Figure S4). In general, considerable degrees of amorphization between 85 and 100% were achieved. On the contrary, an analogous milling of plain ibuprofen lowered the degree of crystallinity just by 4.5% (Supporting Information, Figure S3), as also stated in the literature (Hussain et al., 2018). Consequently, solely the introduced mechanochemical stress is not sufficient to induce a complete and thermodynamically stable transition from the crystalline to the amorphous phase. Therefore, the successful amorphization of ibuprofen within the co-milled blends was achieved as a consequence of a combinatorial impact: the supplied energy by co-milling disrupts the equilibrium structure of crystalline ibuprofen, and the transition to the amorphous state concurrently becomes enhanced by interactions with the SA matrix, as the high adsorption energy of SA overcompensates the energetic advantage associated with the crystalline structure (Rengarajan et al., 2008; Qian et al., 2011). Nonetheless, since the amorphous state is characterized by less favorable thermodynamic properties, such as increased enthalpy, entropy and Gibbs free energy, recrystallization may occur. For that reason, the physical stability of the formulations needs to be considered and investigated over longer periods of storage. Irrespective of the surface properties, the reversion to the crystalline state was utterly inhibited over a storage time of 6 months under ambient conditions for all prepared formulations containing 100% amorphized ibuprofen. Recrystallization is kinetically and thermodynamically driven by molecular mobility and high Gibbs free energy, respectively (Hilden & Morris, 2004). The proven long-term stability allows to conclude that the former was effectively inhibited by confinement of the drug molecules inside the porous host matrix, which becomes even more evident under consideration of the drug release studies in the following section. On the other hand, the structural durability furthermore emphasizes the efficacious reduction of the surface free energy due to intermolecular and surface-defined interactions between the ibuprofen molecules and the terminal functional groups of the SA carrier system. In correspondence, complete amorphization and thus maximum loading capacities were achieved at different mass fractions of ibuprofen for APTES-functionalized (37.0 wt%_ibuprofen_), untreated (27.8 wt%_ibuprofen_) and TMCS-functionalized SA (25.0 wt%_ibuprofen_). Assuming that the adsorption of ibuprofen predominantly occurs via the interactions of the carboxylate group (Alnaief & Smirnova, 2010), hydrophobization by TMCS leads to less active binding sites for the adsorption of ibuprofen resulting in a lower degree of amorphization and thus a higher remaining crystallinity. Interestingly, an increase in the maximum possible drug loading capacity by almost 10% was achieved for SA@APTES compared to the unmodified counterpart SA despite the reduction in surface polarity (Table 1). The mechanochemical stress results in further comminution of the particle fragments, so that new accessible surfaces are generated. As a result, the formation of hydrogen bonds by the amino groups is presumably diminished by the effect of introduced energy. Consequently, these functional moieties are rendered available for the adsorption of ibuprofen, leading to an increased drug loading capacity.

The total amount of amorphized ibuprofen was further increased by applying the melting method (Figure 7B). Accordingly, all prepared formulations initially demonstrate a complete amorphization after the drug loading process. Yet, partial recrystallization occurred above 44.4 wt%_ibuprofen_ for both SA@APTES and plain SA, and at mass fractions exceeding 16.7 wt%_ibuprofen_ regarding SA@TMCS after a storage time of 6 months. In contrast to the co-milled formulations, ibuprofen loses its crystalline structure entirely due to the melting process resulting in an apparently complete amorphization, melted plain ibuprofen however regained 94% of its crystalline structure within 4 weeks (Supplementary Information, Figure S3). Consequently, the presence of a large amorphous fraction of formulations stored for 6 months can certainly be attributed to an effective drug incorporation into the SA network. The increased drug loading efficiency can probably be referred to the preservation of the structural integrity of the SA matrix. Nitrogen sorption analyses of a control experiment showed that the pore volume of unloaded SA decreased by approx. 37% after a second milling procedure (Supplementary Information, Figure S5). Surface functionalization with APTES, however, does not augment the degree of amorphization compared to the bare SA as determined for the co-milled blends. The driving force of the ibuprofen uptake into the porous structure of the SA carrier is primarily dominated here by the acting capillary forces. In fact, supercritically dried SA possesses around 9 wt% of remaining moisture and solvent as determined via thermogravimetric analysis (Supplementary Information, Figure S6). Therefore, the pore walls are strongly contracted and thus the attractive potential exerted by the individual atoms and functional groups at the pore surface overlap and bring about a severe attraction to adsorbate molecules. Despite the fact that no enhanced amorphization was achieved by APTES functionalization, the surface properties have shown to be a governing parameter. Compared to the co-milling approach, loading of TMCS-modified SA was less efficient due to lowered affinity of ibuprofen to the carrier matrix, deteriorating the diffusion into the porous network. Thus, achieving high drug loading efficiencies necessities not only a profound understanding of the effectiveness of the loading process, but also a thorough consideration of the respective surface-related properties.

### In-vitro drug release

**Figure 8.**
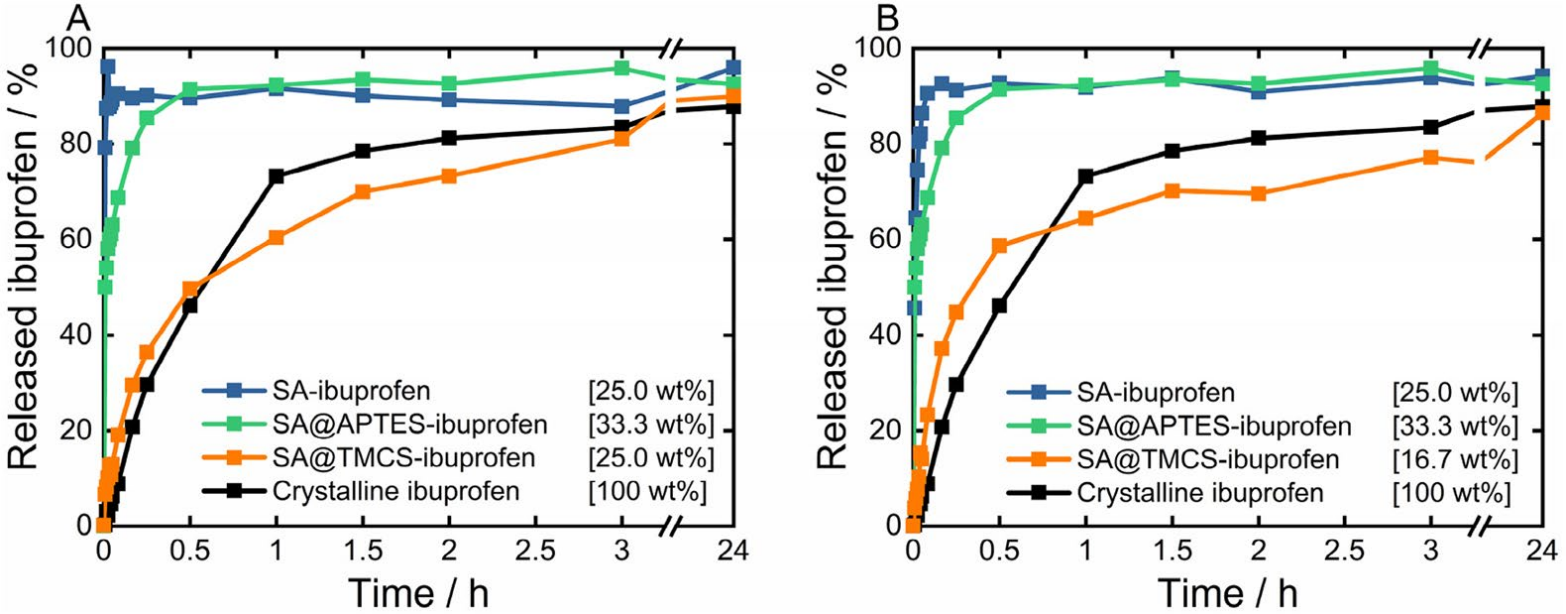
Dissolution profiles of crystalline ibuprofen as a reference as well as release profiles of ibuprofen embedded in plain, APTES- and TMCS-functionalized SA prepared by A) co-milling and B) melting of the physical blends using the USP apparatus II. All investigated ibuprofen-loaded SA formulations solely contained completely amorphized ibuprofen within the respective SA matrix. Yet, the total amount of ibuprofen within all samples was kept constant at 10.8 mg.

Preliminary experiments on the physical stability of the SA carrier system after dispersion in the HCl solution at pH 1.2 revealed that the SA structure was still retained after 24 h dispersion time (Supplementary Information, Figure S7), therefore confirming that ibuprofen is first released from the SA network and consequently dissolved rather than a concurrent dissolution of both substances. To this end, release kinetics of ibuprofen from functionalized and non-functionalized SA are compared to the dissolution rate of crystalline ibuprofen in Figure 8. Herein, samples containing ibuprofen in fully amorphous form were selected for the release studies consequently differing in terms of carrier-to-drug-mixing ratios. However, in previous studies, it was demonstrated that the silica-to-ibuprofen ratio does not influence the release behavior (Melzig et al., 2018b). In general, no considerable differences in the release behavior of the prepared formulations were observed for both drug loading methods. In fact, release profiles obtained for co-milled samples (Figure 8A) were highly similar to profiles observed for formulations processed via the melting method (Figure 8B). Accordingly, the dissolution of ibuprofen from SA@APTES and plain SA formulations occurs in a strongly accelerated way in comparison to the dissolution of pure crystalline ibuprofen. Since these two SA samples maintain their hydrophilicity, the collapse of the SA matrix upon contact with the aqueous medium is not affected. Thus, a fast release and dissolution of amorphous ibuprofen follows: around 80% of ibuprofen dissolves from SA and SA@APTES within 1 and 10 min, respectively, whereas dissolving 80% of crystalline ibuprofen requires 106 min. In contrast, as a result of a slower wetting of the inner surfaces of the SA@TMCS network by the aqueous medium, the release of ibuprofen was substantially delayed despite the fact that ibuprofen was present in the amorphous state. Correspondingly, 80% of ibuprofen was released only after approximately 3 h and 24 h from formulations prepared by co-milling and melting of the blends, respectively. The prolonged release of ibuprofen further confirms the previously drawn assumption that ibuprofen is rather embedded inside the porous SA network than adsorbed on the outer surface of SA particles. The latter would not lead to a sustained dissolution kinetics of SA@TMCS with respect to SA and SA@APTES. Hence, it can be concluded that, depending on the terminal functional entity, an extensive change in the release of the embedded ibuprofen can be achieved even though it is present in the amorphous state in all cases. Thus, surface functionalization with different ligands enables to selectively adjust the ibuprofen release behavior while the drug loading method does not exhibit a significant effect.

## Conclusion

In this work, SA with tailored surface properties were successfully synthesized and the influence of various significant process-related parameters on the resulting material properties elucidated. To this end, the necessity of preparing highly homogeneous and intact SA monoliths was demonstrated. Structural monolithic deformations as a result of erratic supercritical drying lead to deteriorated material properties. This trend was significantly enhanced after a complete collapse of the internal SA network as a result of drying under ambient pressure. A correlated loss of 70% of pore volume emphasized the relevance of a highly controlled drying process under supercritical conditions. Subsequent grinding of monolithic SA was shown to be feasible for powdering the gels without degrading the porous gel network. However, the grinding method of choice and the applied parameters substantially influence the microstructural properties of the obtained SA powders. Accordingly, comminution under cryogenic conditions for 10 min at 15 Hz was shown to provide the most eminent and reproducible specific surface area und porosity values. Moreover, the suitability of the synthesized SA as an amorphizing carrier system for ibuprofen was successfully validated. In order to achieve versatile surface properties and selectively tune the affinity of ibuprofen to the SA matrix, the surface of SA was functionalized with APTES or TMCS. In addition to the surface chemistry, the drug-to-excipient ratio and the post-synthetic drug loading method were identified to be further important determinants in order to achieve high degrees of amorphization. Stability studies of completely amorphized samples revealed an effective inhibition of recrystallization during storage times of at least 6 months. Finally, ibuprofen-loaded SA formulations exhibited tremendously enhanced dissolution rates in comparison to crystalline ibuprofen. Moreover, defined surface functionalization, i.e. the hydrophobization by TMCS, enables to substantially prolong the release and hence dissolution of ibuprofen despite its amorphous state. The possibility to adjust the quantitative release of amorphized APIs is highly advantageous not only to achieve immediate relief of acute pain as in the use of nonsteroidal anti-inflammatory drugs, such as ibuprofen, but also for prolonged drug release, e.g. for effective regenerative processes in the field of tissue engineering. To this end, coating of SA could furthermore be performed with hydrophilic organics such as polyethylene glycol or the pH responsive hydroxypropyl methylcellulose, to potentially achieve a site-specific and controlled release, e.g. hydrophilic drugs over an extended period of time. These diverse possibilities of tailoring the SA surface properties, the drug-independent synthesis procedure as well as the flexibility of the post-synthetic drug loading strategies render the presented strategy highly promising for incorporation and stabilization of many other drug compounds with different hydrophobicity. In essence, the adaptation of the surface chemistry and an in-depth knowledge about the relevant process parameters offer versatile possibilities for the selective preparation of physically stable drug-SA formulations with tunable therapeutic properties.

## Supplementary Material

Supplemental Material

## Abbreviations

API: active pharmaceutical ingredients
APTES: (3-aminopropyl)triethoxysilane
BET: Brunauer, Emmett, and Teller
BJH: Barrett, Joyner, and Halenda
CPD: critical point dryer
CVBM: cryogenic-vibratory ball mill
DSC: dynamic scanning calorimetry
MID: multiple-ion detection
MS: mass spectrometry
MO: mortar
SA: silica aerogels
SAXS: small-angle X-ray scattering
scCO2: supercritical carbon dioxide
SEM: scanning electron microscopy
SS: sieve shaker
TGA: thermogravimetric analysis
TMCS: trimethylchlorosilane
TMOS: tetramethyl orthosilicate
VBM: vibratory ball mill
