# Supplementary material for: Amorphization and modified release of ibuprofen by post-synthetic and solvent-free loading into tailored silica aerogels
Source: Drug Deliv. 2022 Jul 15;29(1):2086–99. doi: 10.1080/10717544.2022.2092237 (PMC9291651; doi:10.1080/10717544.2022.2092237)
Supplement: Supplemental Material [file IDRD_A_2092237_SM7639.docx]

# Supplementary Information

**Amorphization and Modified Release of Ibuprofen by Post-Synthetic and Solvent-Free Loading into Tailored Silica Aerogels**

*Ajmal Zarinwall^1,2^, Viktor Maurer^1,2^, Jennifer Pierick^1,2^, Victor Oldhues^1,2^, Julian Cedric Porsiel^1^, Jan Henrik Finke^1,2^, Georg Garnweitner*^1,2^*

^1^ Institute for Particle Technology (iPAT), Technische Universität Braunschweig, 38104 Braunschweig, Germany

^2^ Center of Pharmaceutical Engineering (PVZ), Technische Universität Braunschweig, 38106 Braunschweig, Germany

* Correspondence: g.garnweitner@tu-braunschweig.de

**Table of content**

1. Additional technical details
2. Figure S1: SEM images of differently ground SA
3. Figure S2: Elemental analysis of APTES- and TMCS-functionalized SA
4. Figure S3: DSC measurements of untreated, co-milled and melted ibuprofen
5. Figure S4: DSC studies of co-milled and melted ibuprofen-SA formulations
6. Figure S5: Nitrogen sorption analysis of SA after comminution processes
7. Figure S6: TGA profiles of APTES-modified SA
8. Figure S7: SEM images of SA after dispersion in 0.1 M HCl solution.
9. Table S1: Varied parameters throughout the milling studies.
10. Table S2: Overview of parameter study using the VBM.

**Additional technical details**

**Thermogravimetric analysis (TGA)** was carried out on a TGA/DSC 1 STARe system and a gas controller 4C200 STARe system from Mettler Toledo GmbH (Columbus, Ohio, USA). 10-15 mg of the dried sample was heated at a rate of 10 °C/min under an oxygen atmosphere.


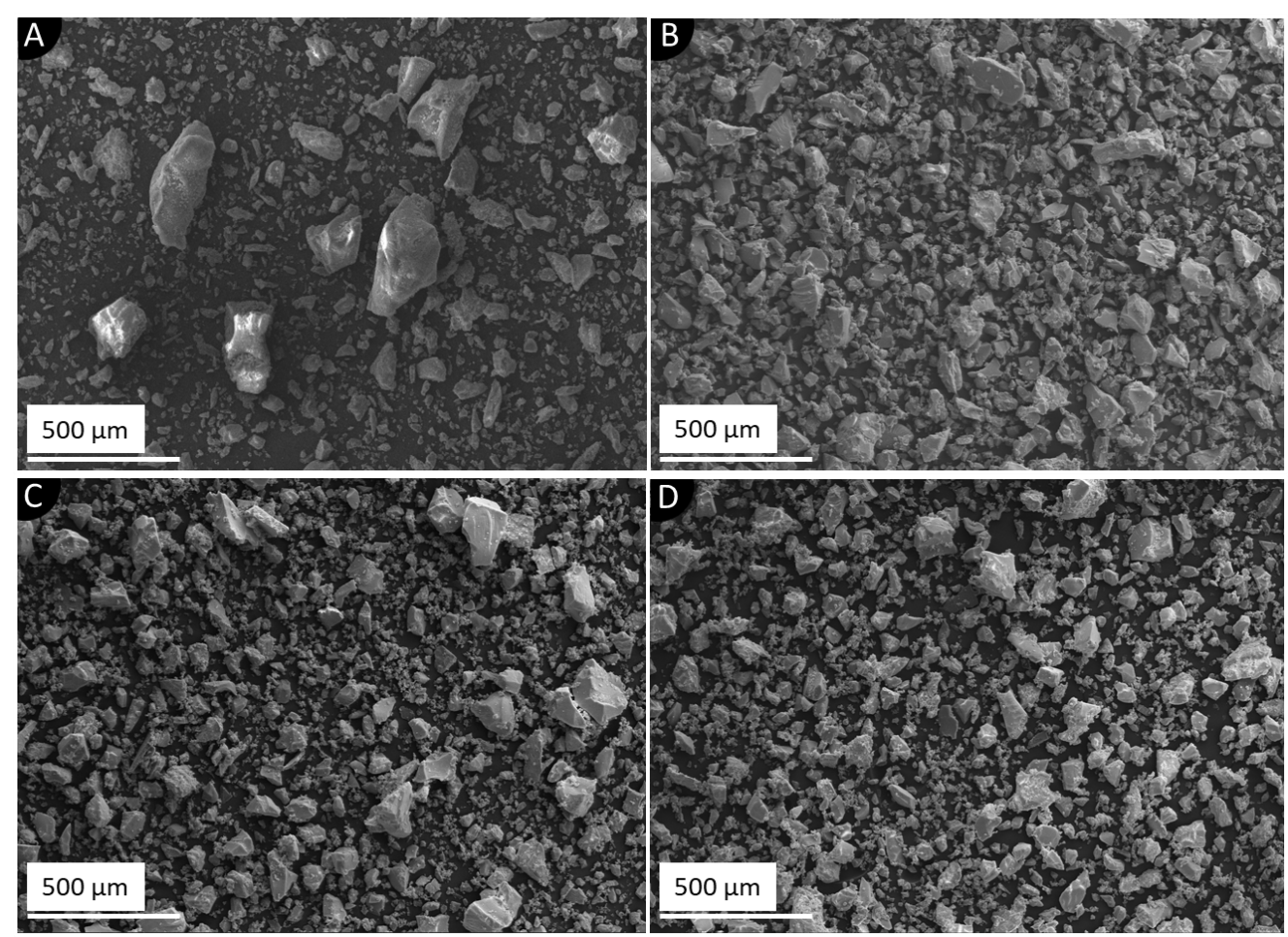


**Figure S1.** SEM images of SA after A) grinding by mortar, B) subsequent classification via sieving, and milling with a vibratory ball mill under C) ambient and D) cryogenic conditions.


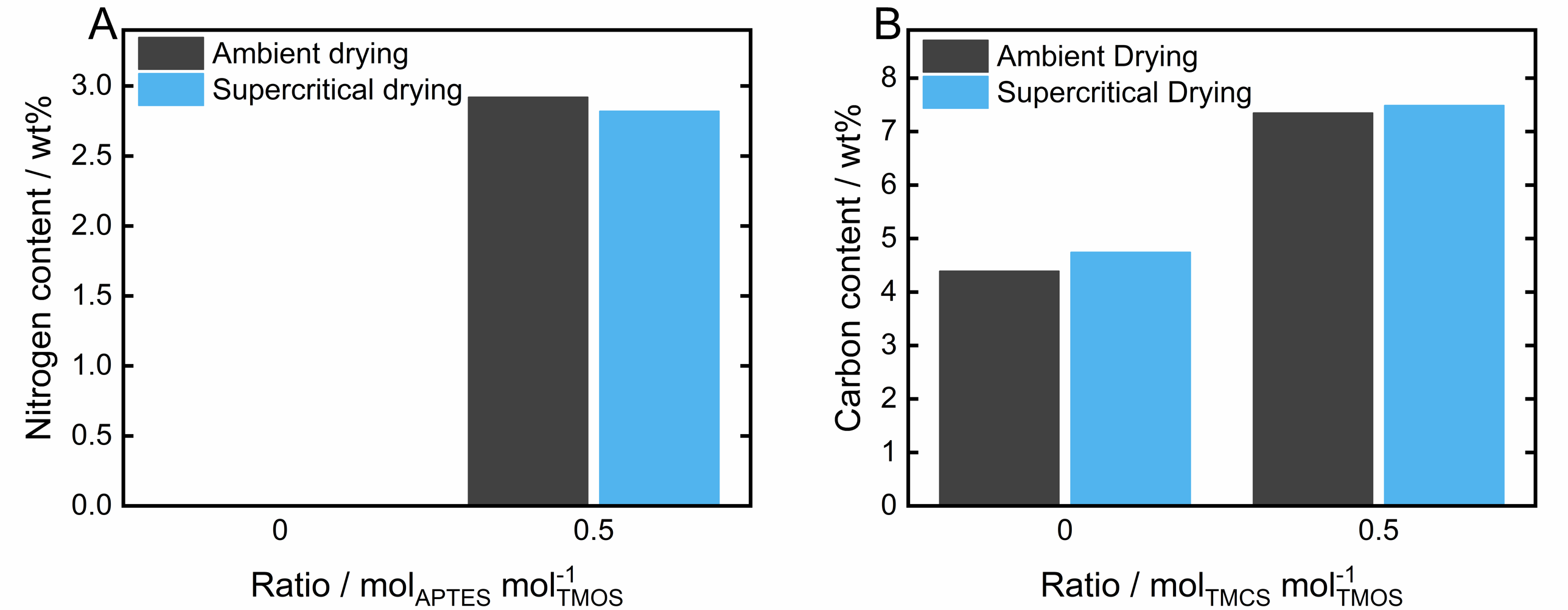


**Figure S2.** Influence of the drying process on the resulting amount of bound A) nitrogen corresponding to APTES and B) carbon corresponding to TMCS on the SA, determined via elemental analysis. Each functionalization was carried out for 24 h.


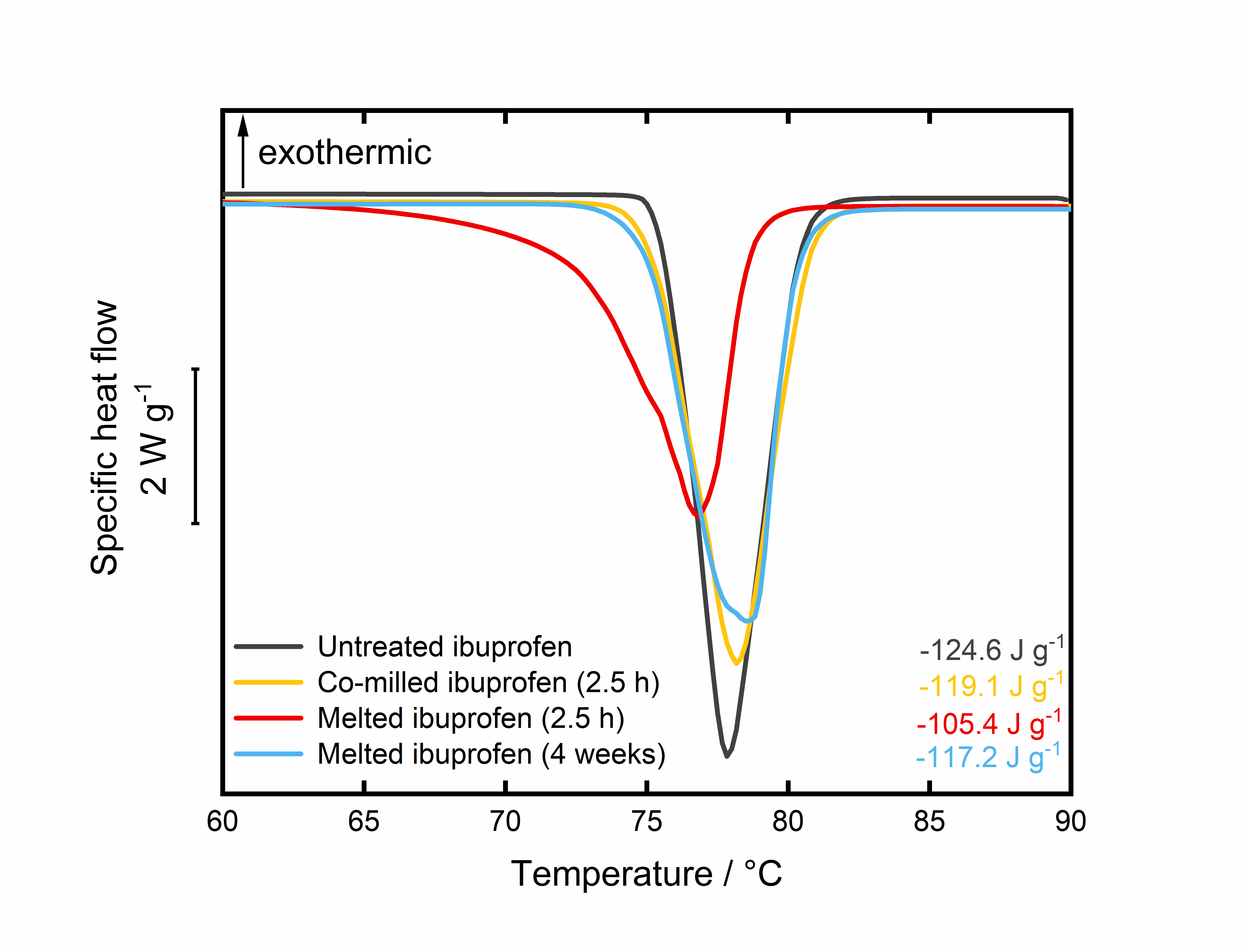


**Figure S3.** DSC measurements of untreated, co-milled and melted ibuprofen. The co-milling approach was carried out solely with bare ibuprofen for 15 min, 15 Hz and at room temperature using 6 grinding balls. Since a higher loss of crystallinity was detected for melted ibuprofen, a second measurement after 4 weeks was carried out.


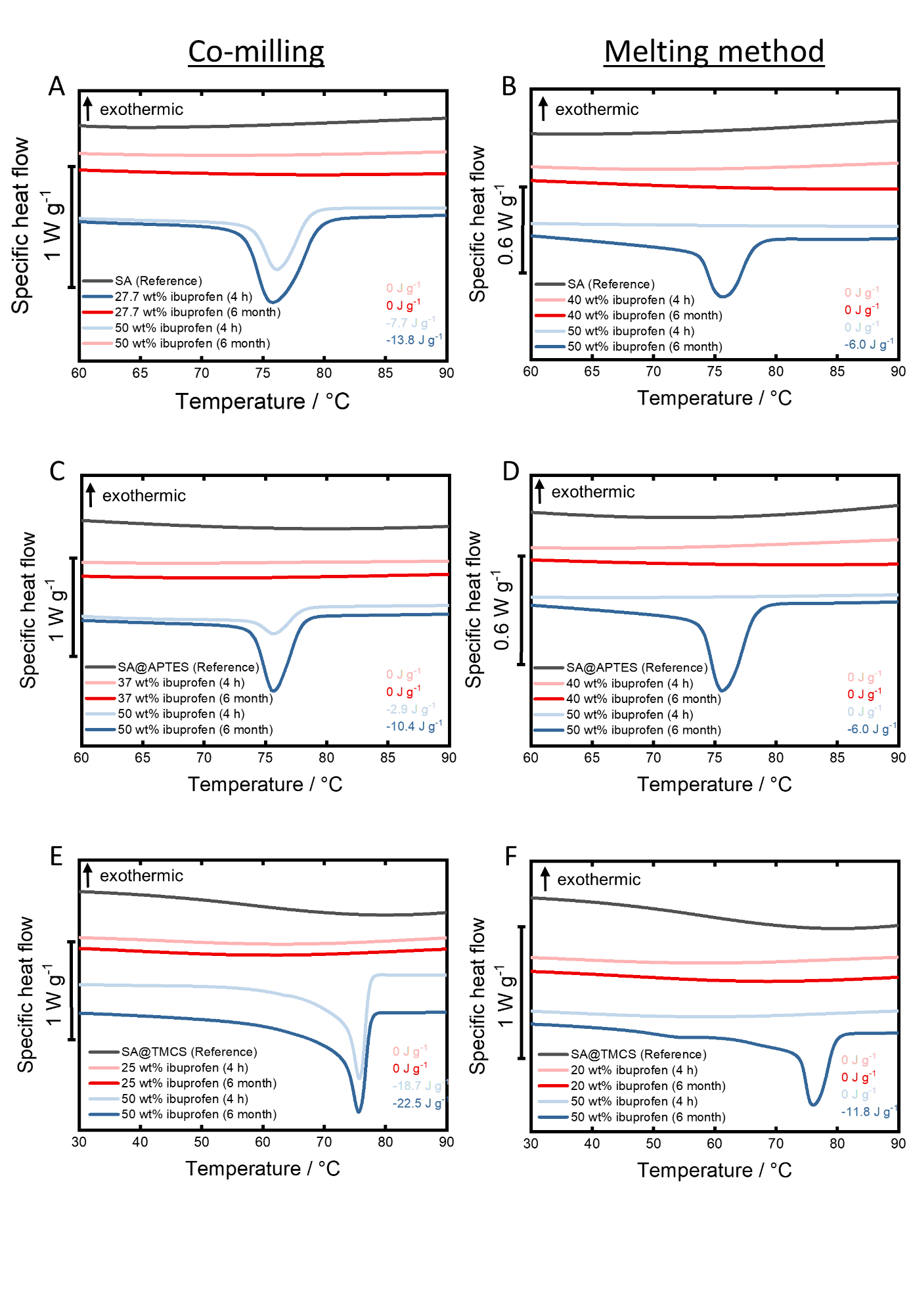


**Figure S4.** DSC studies of prepared ibuprofen-SA formulations. Individual blending ratios were chosen as examples from which a permanent amorphous state was retained even after a storage time of 6 months, as well as mixtures with partially crystalline ibuprofen fractions: Diagrams on the left (A, C, E) illustrate the DSC curves obtained after co-milling of SA-, SA@APTES- and SA@TMCS-ibuprofen mixtures, respectively. On the right-hand side (B, D, F) DSC curves of the blends after application of the melting method are shown. Furthermore, the correlating enthalpy of fusion of ibuprofen of each formulation is depicted.

**
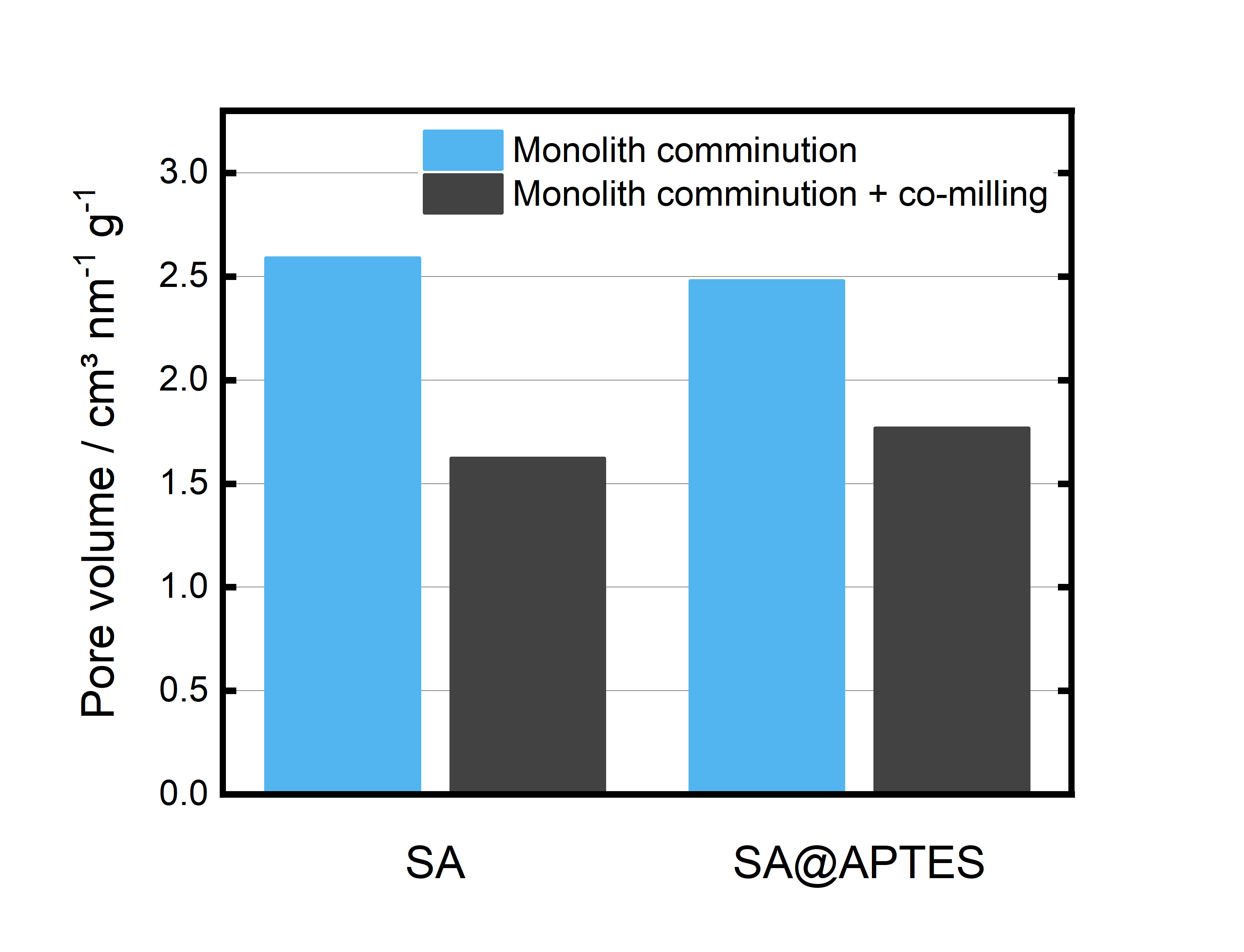
**

**Figure S5.** Influence of a second (co-)milling process on the pore volume of SA and SA@APTES (0.5 mol_APTES_ per mol_TMOS_). Both the comminution and the co-milling were carried out each for 15 min at 15 Hz and room temperature.

**
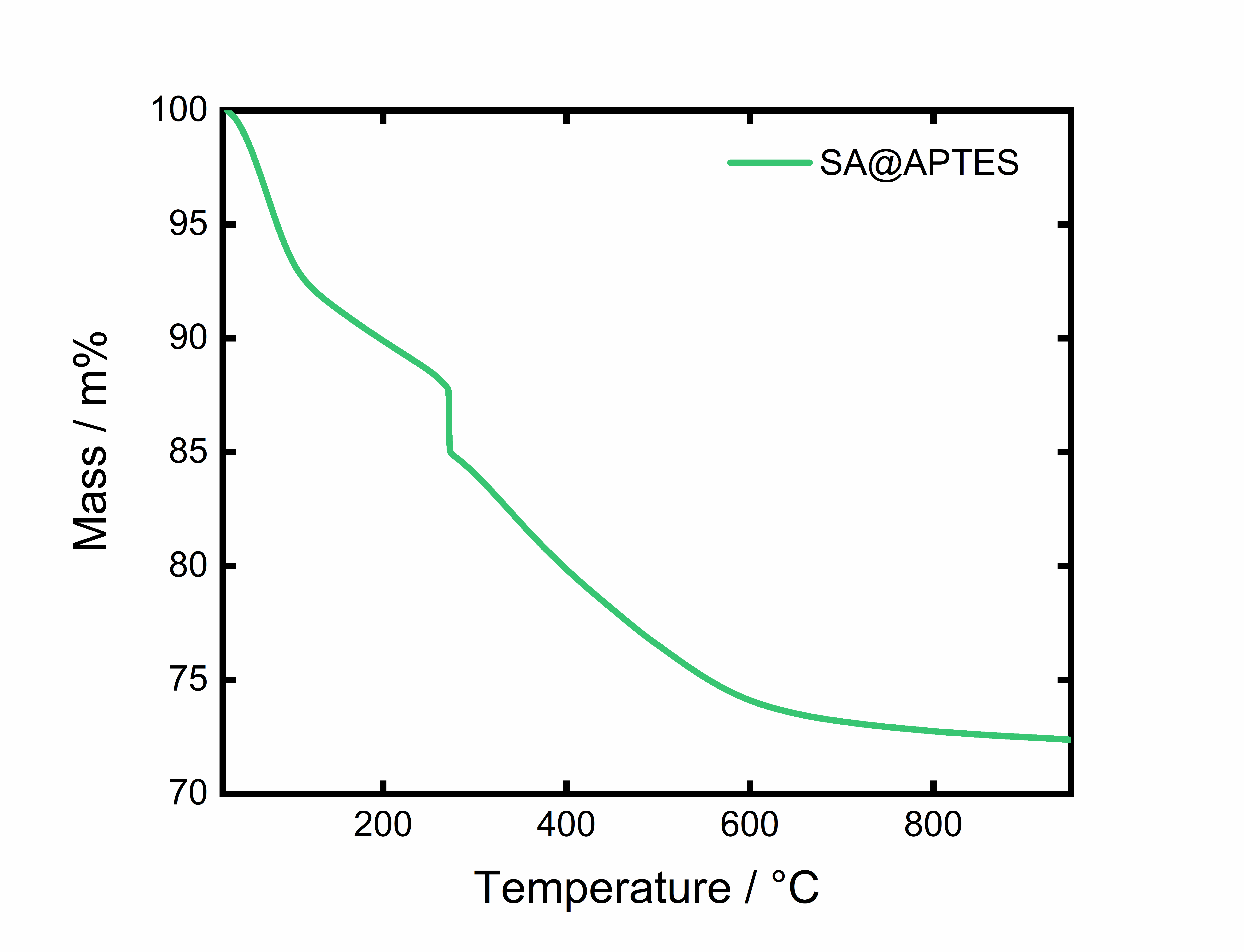
**

**Figure S6.** TGA of SA@APTES. APTES functionalization was carried out with a molar ratio of 0.5 mol_APTES_ per mol_TMOS_ for 24 h at room temperature.

**
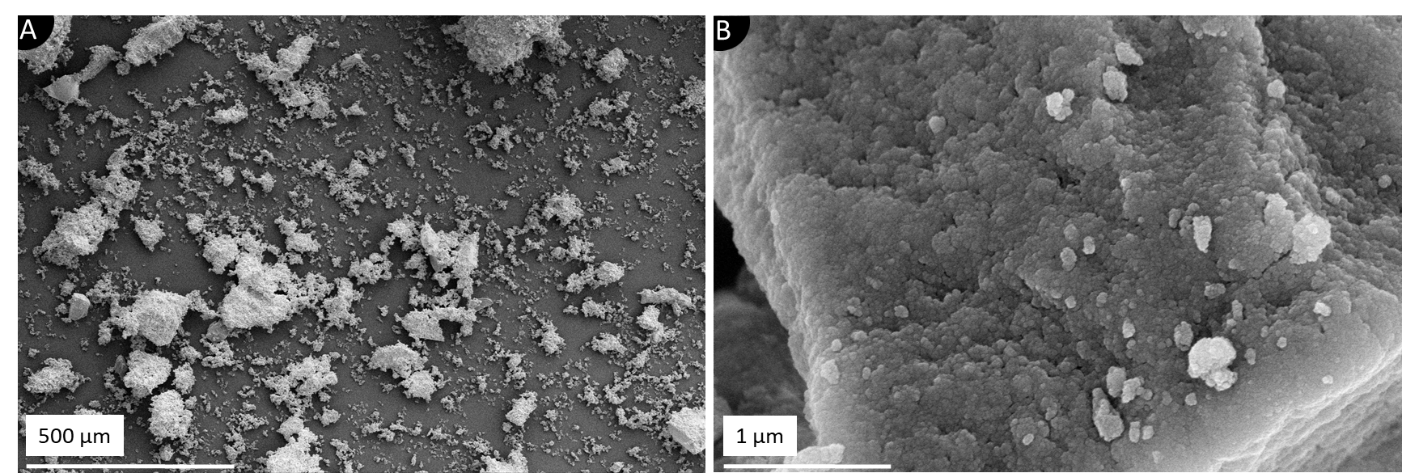
**

**Figure S7.** SEM images of ground SA with A) 250x and B) 100,000x magnification. SA powder was previously dispersed in 0.1 M HCl solution (pH 1.2) for 24 h and then dried under ambient conditions for 48 h at 80 °C.

**Table S1.** Varied parameters throughout the milling studies using the vibratory ball mill at ambient temperature (VBM) and under cryogenic conditions (CVBM).

| Sample | Milling time / min | Frequency / Hz |
| --- | --- | --- |
| VBM10-15 | 10 | 15 |
| VBM15-15 | 15 | 15 |
| VBM10-20 | 10 | 20 |
| VBM15-20 | 15 | 20 |
| VBM5-30 | 5 | 30 |
| CVBM10-15 | 10 | 15 |
| CVBM15-15 | 15 | 15 |
| CVBM10-20 | 10 | 20 |
| CVBM15-20 | 15 | 20 |

**Table S2.** Overview of parameter study using the VBM at room temperature and under cryogenic conditions.

|  | **VBM** | | | | | **CVBM** | | | |
| --- | --- | --- | --- | --- | --- | --- | --- | --- | --- |
|  | VBM  10-15 | VBM  15-15 | VBM  10-20 | VBM  15-20 | VBM  5-30 | CVBM  10-15 | CVBM  15-15 | CVBM  10-20 | CVBM  15-20 |
| *S*_A_ /  m² g^-1^ | 676.9 | 976.7 | 807.6 | 695.9 | * | 982.5 | 767.9 | 914.3 | 760.7 |
| V_P_ /  cm³ g^-1^ | 1.8 | 2.4 | 1.5 | 1.1 | * | 3.2 | 2.4 | 2.3 | 1.8 |
| d_pores_ / nm | 9.3 | 8.7 | 6.6 | 5.5 | * | 11.6 | 11.0 | 9.4 | 8.3 |
| d_particles_ / μm | 145.1 | 137.2 | 143.2 | 145.5 | * | 118.5 | 171.3 | 154.0 | 158.8 |

* Due to intensive grinding media abrasion, no valid measurements were possible.

The specific surface area and the pore structure of the investigated SA differed significantly for differently applied parameters. Grinding by the use of a VBM ensues as a result of the inertia of grinding balls, that causes them to impact with high energy on the SA. Thus, comminution by means of impact stress entails the risk of structural deformation of the internal gel network. If not operated efficiently, part of the introduced energy results in plastic deformation or compression of the open pore structured SA instead of efficient particle breakage as determined for milling processes (VBM and CVBM) with a frequency of 20 Hz that resulted in a significant loss of pore volume and -size. In addition, a slight grey haze was visually identified in these powdered samples as a result of grinding media abrasion. The contamination of the sample material was substantial at a frequency of 30 Hz (VBM5-30), so that no reliable analyses were feasible. Nonetheless, the greatest possible energy utilization for particle breakage was achieved by drastically lowering the temperature to -196 °C (CVBM10-15). Exploitation of SA embrittlement at low temperature enabled the most effective preservation of the porous nature. Thus, the largest specific surface area (982.5 m²/g), pore diameter (11 nm) and pore volume (3.2 cm³/g) were obtained for milling under cryogenic conditions for 10 min at 15 Hz, indicating the highest grinding efficiency. Considering the characteristics of the collapsed SA (Fig. 3B), it can be concluded that an effective comminution of the macrostructure could be achieved while maintaining the aerogel-typical highly porous microstructure.
